# Instantaneous transport of a passive scalar in a turbulent separated flow

**DOI:** 10.1007/s10652-017-9567-3

**Published:** 2017-12-18

**Authors:** P. Ouro, B. Fraga, N. Viti, A. Angeloudis, T. Stoesser, C. Gualtieri

**Affiliations:** 1 Hydro-environmental Research Centre, School of Engineering, Cardiff University, The Parade, Cardiff, CF24 3AA UK; 2 Applied Modelling and Computation Group, Department of Earth Science and Engineering, Imperial College, London, UK; 3 Civil, Architectural and Environmental Engineering Department (DICEA), University of Napoli “Federico II”, Naples, Italy; 4 School of Civil Engineering, University of Birmingham, Edgbaston, Birmingham, B15 2TT UK

**Keywords:** Large eddy simulation, Separation flows, Solute dispersion, Backward facing step, Turbulence

## Abstract

The results of large-eddy simulations of flow and transient solute transport over a backward facing step and through a 180° bend are presented. The simulations are validated successfully in terms of hydrodynamics and tracer transport with experimental velocity data and measured residence time distribution curves confirming the accuracy of the method. The hydrodynamics are characterised by flow separation and subsequent recirculation in vertical and horizontal directions and the solute dispersion process is a direct response to the significant unsteadiness and turbulence in the flow. The turbulence in the system is analysed and quantified in terms of power density spectra and covariance of velocity fluctuations. The injection of an instantaneous passive tracer and its dispersion through the system is simulated. Large-eddy simulations enable the resolution of the instantaneous flow field and it is demonstrated that the instabilities of intermittent large-scale structures play a distinguished role in the solute transport. The advection and diffusion of the scalar is governed by the severe unsteadiness of the flow and this is visualised and quantified. The analysis of the scalar mass transport budget quantifies the mechanisms controlling the turbulent mixing and reveals that the mass flux is dominated by advection.

## Introduction

The accurate reproduction of mixing processes over a range of flow conditions where turbulence plays an important role in both hydrodynamics and scalar transport is a fundamental research challenge of environmental fluid mechanics. In turbulent flows there is a wide range of flow structure scales which characterise the flow dynamics [42]. The large-scale hydrodynamic structures determine the global flow pattern. Meanwhile, small-scale structures are intermittently present and play an important role in mixing processes as they can accelerate the diffusion of substances within the flow [48]. In this study, the instantaneous resolution of the flow field is predicted via large-eddy simulations that permit an analysis of a passive scalar’s dispersion in an irregular geometry. This facilitates a visualisation and characterisation of the scalar’s transport and the flow’s turbulence nature in a flow over a backward facing step and the 180° bends of a multi-compartment channel geometry.

Flow separation occurs when a portion of the flow departs from the main streamwise direction to form secondary currents and vortices that can influence interconnected processes beyond the primary hydrodynamics. It is characterised by a well-defined separation streamline that divides the flow into two non-communicating regions [11]. Examples of its occurrence include the deflection of submerged water jets against solid boundaries, sharp curvatures in meandering river bends [9, 11, 13, 21], abrupt changes in the streamwise flow bed [15, 45, 49], flow over a dune [44], massively separated flows over a step [20, 25] and extend to engineering applications beyond the strict context of environmental fluid mechanics (e.g. airfoil design).

A passive scalar can be defined as an idealised non-reactive substance that is transported along the volume of fluid featuring negligible interference with the local hydrodynamics. The transport of active or passive scalar quantities within a given flow domain is governed by advective and diffusive processes where turbulence plays a key role in their mixing and distribution. In fact, processes such as the transport of pollutants or the injection of a tracer [7, 8] are often treated as passive scalar quantities. Understanding how a turbulent separated flow impacts the transport of a passive scalar can be of particular interest in environmental applications such as sediment deposition, channel scour [27], sidewall erosion, tracer dye transport [3, 4] and pollutant dispersion [16]. Therefore, the dispersion of passive scalars in turbulent flows has been at the forefront of environmental fluid mechanics research as demonstrated by several pioneering studies, e.g. [40].

Over the last 2 decades, computational fluid dynamics (CFD) has been employed to simulate scalar transport processes in conjunction with experimental campaigns, thus informing environmental fluid mechanics studies. These models have been predominantly based on the solution of the Reynolds Averaged Navier–Stokes (RANS) equations [2, 17, 36, 50]. RANS modelling is appropriate when focus is on time-averaged flow properties. A turbulence model is incorporated to provide a closure to the calculation of the Reynolds stresses. This is accompanied with certain assumptions such as the turbulence isotropy when an eddy-viscosity formulation is imposed. For example, this is applicable to the standard versions of the $k-\varepsilon$ and $k-\omega$ models. RANS approaches have been extensively used in research and practical engineering for environmental fluid mechanics applications due to their relatively low computational cost and ability to produce accurate results for time-averaged quantities. However, RANS models struggle when unsteady, large-scale flow structures dominate the flow and transport processes [42].

The method of large-eddy simulation (LES) simulates directly (most of) the instantaneous flow field allowing a more comprehensive analysis of the transient hydrodynamics and by extension scalar transport. In LES, large-scale flow structures are resolved and there are no turbulence models in the same context as RANS; with the latter prone to further uncertainty in the results as discussed in [51]. On the contrary, in LES a sub-grid scale (SGS) model is introduced in LES that accounts for the small-scale turbulence smaller than the filter size, in most cases the mesh size [38]. The main drawback of LES relates to the fine meshes required to accurately resolve all relevant temporal and spatial scales of the flow at the expense of increased computational time. Nevertheless, the exponentially increasing availability of high performance computing resources [39] gradually makes LES accessible and practical among several fluid mechanics fields [42].

In the study reported here, LES is employed to provide insights into the influence of instantaneous turbulence on passive scalar transport within a particular three-dimensional separated flow, which may be described as a backwards facing step. The numerical methodology is presented in Sect. 2 together with the details of the chosen flow configuration. Section 3.1 shows the hydrodynamic validation of the method using experimental data in terms of time-averaged velocities and analyses the turbulence nature within the separated flow via quadrant analysis and power spectral density. Analysis of the scalar transport is described in Sect. 3.2, focusing on the impact of the instantaneous flow field on the mixing, contribution of mass fluxes into the scalar transport budget, and comparison of scalar concentration curves between the computed results and experiments. Finally, Sect. 4 provides the main conclusions drawn from this study.

## Methodology

### Hydrodynamics and scalar transport simulations

The fluid flow is simulated using the in-house LES solver Hydro3D which has been validated in a number of complex flows studies including tidal current turbines [33, 35], bubble plumes [12, 14] and many other hydraulic applications [6, 29, 41, 43]. The governing equations are the spatially filtered Navier–Stokes equations for turbulent, incompressible, three-dimensional flow which read:

1 $$\begin{aligned}&\frac{\partial u_i}{\partial x_i} =0 \end{aligned}$$

2 $$\begin{aligned}&\frac{\partial u_i}{\partial t} + \frac{\partial u_i u_j}{\partial x_j} = - \frac{\partial p}{\partial x_j} + \nu \frac{\partial ^2 u_i}{\partial x_i x_j} + f_i \end{aligned}$$

where $u_i$ and $x_i$ are the fluid velocities and coordinates in the three directions in space (*i* or *j* = 1, 2, 3) respectively, *p* is the pressure and $f_i$ is a source term representing forces from the immersed boundary (IB) method. The total viscosity, $\nu =\nu _l+\nu _{sgs}$, results from the addition of the fluid kinematic viscosity, $\nu _l$, and the turbulent viscosity obtained from the sub-grid scale (SGS) model, $\nu _{sgs}$. The latter is computed using the Smagorinsky [38] SGS model, where the constant $C_S$ is set constant with a value of 0.1, as it has been proved to provide successful predictions in scalar transport [24, 28].

The derivatives are approximated with fourth-order central scheme finite differences with staggered storage of the velocity components on a Cartesian rectangular grid and the fractional-step method [10, 47] summarised in Eqs. 3–7.

3 $$\begin{aligned} \frac{\tilde{u_i}-{u_i}^{t-1} }{{\Delta t} }&= \alpha _k {\mathbb {C}} {u_i}^{k-1} +\beta _k {\mathbb {C}} {u_i}^{k-2} + \frac{1}{2}\alpha _k {\mathbb {D}} \left( {u_i}^{k-2}+{u_i}^{k-1}\right) - \alpha _k \frac{\partial p^{t-1}}{\partial x} \end{aligned}$$

4 $$\begin{aligned} {\tilde{u_i}}^{*}&=\tilde{u_i} + f_i \Delta t \end{aligned}$$

5 $$\begin{aligned} {\nabla }^2 \tilde{p}&= \frac{\nabla \tilde{u_i}^{*}}{(\alpha _k + \beta _k) \Delta t} \end{aligned}$$

6 $$\begin{aligned} u_i&= {\tilde{u_i}}^{*} - \alpha _k \Delta t \nabla \tilde{p} \end{aligned}$$

7 $$\begin{aligned} p&= {p}^{t-1} + {\tilde{p}} - \frac{\alpha _k \nu \Delta t}{2} \nabla ^2 \tilde{p} \end{aligned}$$

First in Eq. 3 the predicted fluid velocity ${\tilde{u_i}}$ is calculated from a combination of a third-order low-storage Runge–Kutta and Crank–Nicholson methods for convective and diffusive terms respectively. The Runge–Kutta coefficients are $\alpha _k=\beta _k$ = 1/3, 1/2 and 1 where *k* indicates the Runge–Kutta step, *t* is the time variable and ${\mathbb {C}}$ and ${\mathbb {D}}$ denotes convection and diffusion operations respectively. The predicted velocity is updated in Eq. 4 with the volume force from the IB method to obtain ${\tilde{u_i}^{*}}$. The solution of the Poisson pressure-correction equation (Eq. 5) is achieved using multi-grid technique to obtain the pseudo-pressure ${\tilde{p}}$ used in Eq. 6 as a corrector to obtain the final velocities, $u_i$. Finally, the pressure *p* is obtained through Eq. 7.

The representation of the interior walls that divide the compartments of the simulated backward facing step is accomplished using a refined version of the direct forcing IB method developed by Uhlmann [46] that has been validated in complex fluid-structure interaction problems, as in [26, 33]. This IB method allows the representation of solid obstacles within the fluid domain without the need for building body conformal meshes. Although the IB method can be used in unstructured meshes [34], it is commonly used with rectangular Cartesian meshes in conjunction with fast Poisson equation solvers, such as the multi-grid method, which makes LES more feasible as the computational performance is improved, i.e. by reducing the computational resources. Hydro3D is parallelised through the message passing interface (MPI) that allows the subdivision of the fluid domain in rectangular regions via domain decomposition.

The transport of a passive scalar is simulated in an Eulerian framework by solving a filtered advection–diffusion equation. The filtered scalar transport equation is solved at each time step once the fluid field is calculated, and reads,

8 $$\begin{aligned}&\frac{\partial C}{\partial t} + {u_i} \frac{\partial C}{\partial x_i} = (D+D_t) \frac{\partial ^2 C}{\partial x_i^2} \end{aligned}$$

where *C* denotes the scalar or tracer concentration, $D_t={\nu _t}/{Sc_t}$ is the sub-grid scale turbulent diffusivity and *D* is the molecular diffusivity. The molecular mass flux is deemed negligible in comparison with the turbulent fluxes [22]. $Sc_t$ represents the turbulent Schmidt number that has a value of 0.7 as adopted in similar studies [18, 28].

### Case study and experimental data

**Fig. 1:**
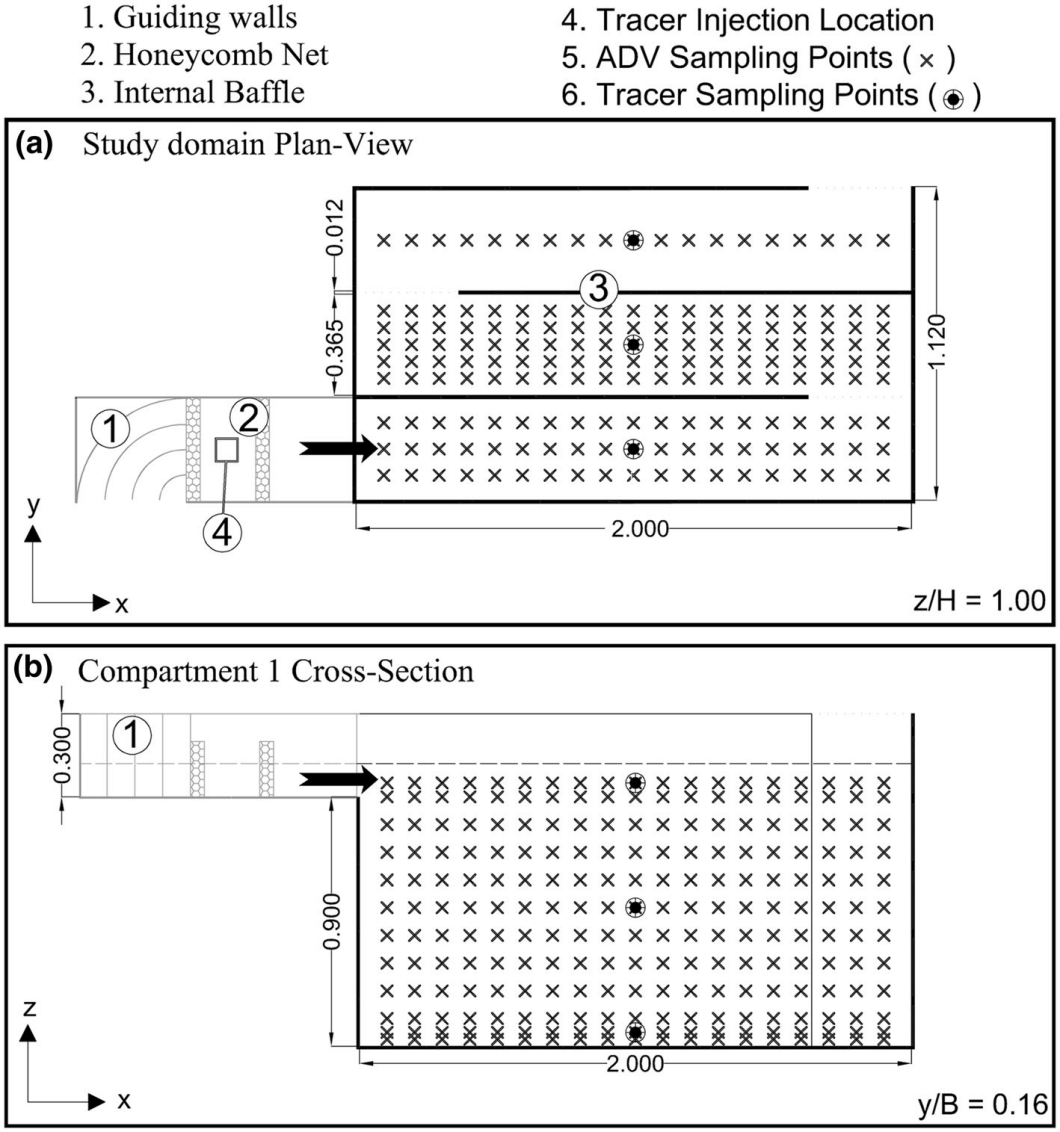
Numerical model geometry and the main sampling points, P1–P6, at which tracer dye data were collected

**Fig. 2:**
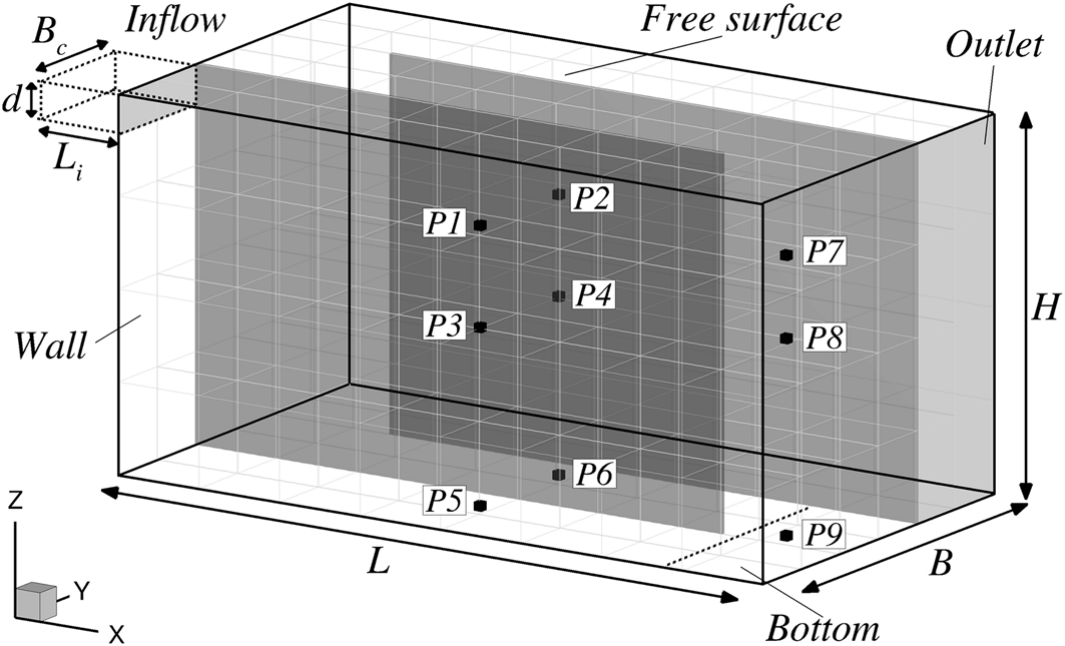
Three dimensional representation of the numerical domain, description of boundary conditions used and specification of the nine reference points at which data time series are obtained for tracer and turbulence analysis

Numerical simulations are validated with the experimental data from [4, 5] obtained from an investigation of contact tank hydrodynamics and disinfection processes. The general layout of the first three compartments of the tank is shown in Fig. 1. Acoustic Doppler Velocimeter (ADV) data were collected at various locations to characterise the hydrodynamics and turbulence behaviour of the multi-compartment tank. In addition, a series of Rhodamine dye tracer experiments were carried out and concentration data were sampled at several locations (P1–P6) as indicated in Fig. 2. The locations are in the centre of each compartment along the streamwise and spanwise directions, i.e. *x*/*L* = 0.5 and *y*/*B* = 0.16 and 0.50, and at variable depths with P1 and P2 located at *z*/*H* = 0.772, P3 and P4 at *z*/*H* = 0.55, and P5 and P6 at *z*/*H* = 0.024.

In the experiment the flow enters the tank through a channel outfitted with a honeycomb net to promote inflow uniformity, and it features a designated mechanism for tracer injections (Fig. 1). Although the experimental tank comprised 8 compartments, the present numerical study focuses on the hydrodynamics developed along the first two compartments, as illustrated in Fig. 2. The domain of interest is $L = 2.0$ m long and $B = 1.12$ m wide and each compartment has a width of $B_c = 0.365$ m, with the intermediate walls being 12 mm thick. The water depth was measured as $H = 1.014$ m. The approach flow had a discharge rate of* Q* = 0.00472 m^3^/s and the inlet area was $d = 0.114$ m deep and 0.365 m wide (Fig. 1). The average cross-sectional inlet velocity was $U_0=0.113$ m/s.

The computational domain is geometrically identical to the experiment but includes only three compartments. The free surface is approximated as a shear-free rigid lid while no-slip conditions are imposed on the side and bottom walls of the channel and the internal baffles are represented by the IB method. The outlet is at the exit of the third compartment and a Neumann boundary condition $(\partial u/\partial x=0)$ is used.

The honeycomb in the experiment entails some uncertainty with regards to the inflow condition and thus two different inflow boundary conditions are tested in order to investigate their appropriateness in simulating the experimental inflow channel. The first approach uses a prescribed 1/7th power-law velocity distribution in both vertical and horizontal directions at the domain inlet face, which takes into account the developed boundary layers in the inflow channel. The second approach comprises a precursor channel (see Fig. 2) that is $L_i = 0.48$ m long and at the inlet of which a uniform velocity distribution is prescribed in an attempt to mimic the effect of the honeycomb. The slowly developing boundary layer flow and turbulence at the outlet of the precursor channel is being fed into the main domain. The Froude number at the inlet channel is $Fr=U_0/\sqrt{gd}\approx 0.11$ that is low enough to consider valid the assumption of modelling the free surface as a frictionless lid [1, 19, 31].

The computational domain, presented in Fig. 2, is divided into 192 MPI sub-domains and the mesh is uniform across its entirety. Two simulations at different mesh resolution are performed and Table 1 specifies the details of the two meshes, namely mesh I and mesh II, including the number of grid points $(n_{x_i})$ and grid spacing $(\Delta x_i)$ in each spatial direction, the total number of grid cells $(N_E)$, number of CPUs and the total time to run each simulation. The simulation using mesh I is carried out on a workstation using 12 Intel Xeon X5650 cores at 2.6 GHz while the simulation using mesh II run in Cardiff University’s supercomputer Raven using 64 Intel Xeon (Sandy Bridge) E5-2670 at 2.6 GHz processors.

**Table 1:** Details of the mesh resolution and computational resources used

Mesh	$\Delta$x	$\Delta$y	$\Delta$z	$n_x$	$n_y$	$n_z$	$N_E$	CPUs	Run time (h)
I	0.008	0.009	0.008	248	120	128	3,809,280	12	72
II	0.004	0.004	0.004	496	270	256	34,283,520	64	144

**Fig. 3:**
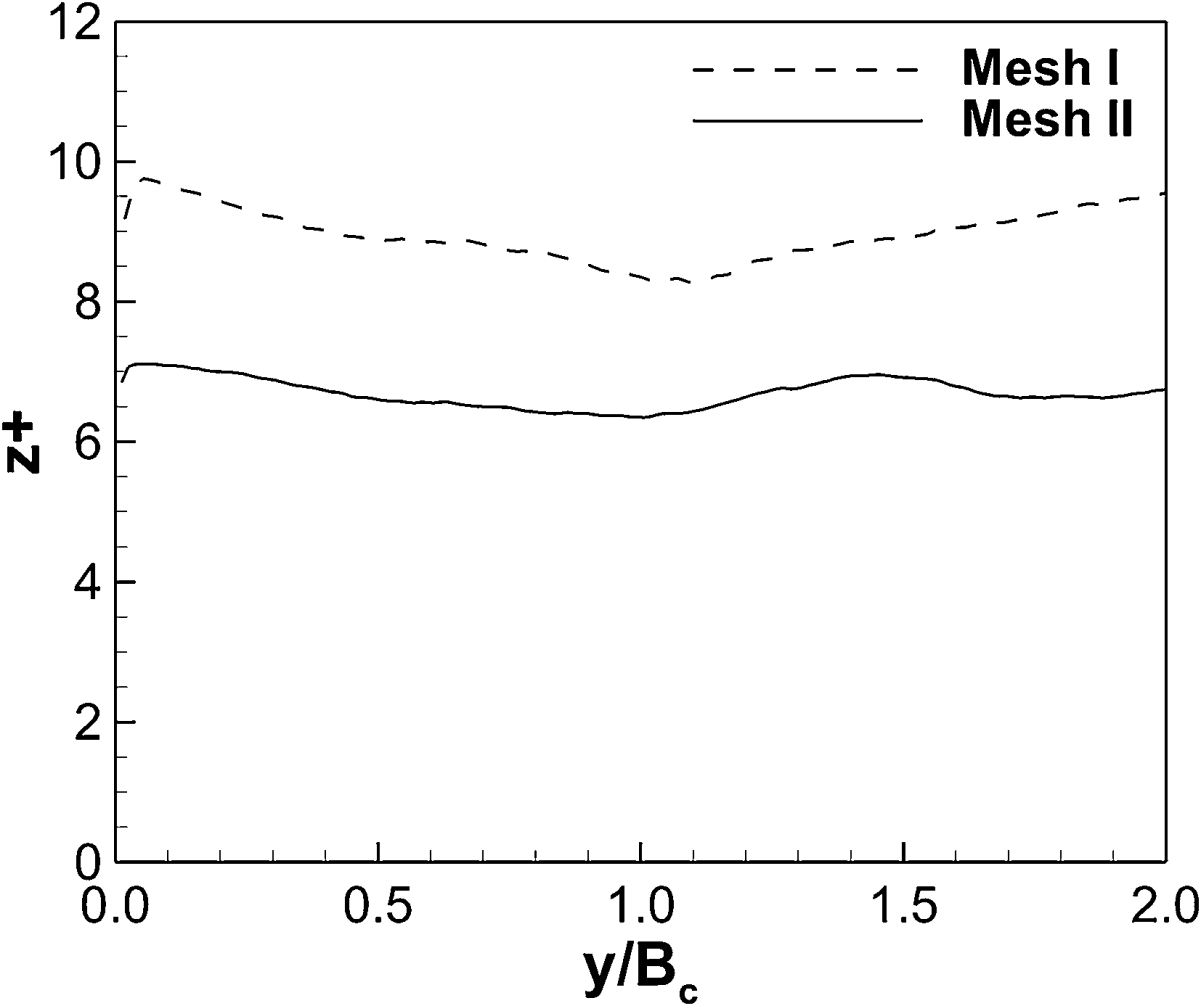
Wall-adjacent grid spacing in wall units along the bottom wall at $x/L=0.8$ along $y/B_c\le 2.0$ for the two meshes used in the simulations

The eddy turn-over time of the first compartment is estimated to be $t_e=L/U_b \approx 150$ s, where $U_b$ is the cross sectional bulk velocity $(U_b=Q/(H B_c)\approx 0.0127)$ m/s. The simulations are initially run for 400 s $(\approx 2.67t_e)$ before averaging of velocities commenced and second order statistics are collected and computed 300 s $(\approx 2.0t_e)$ after that. The total simulation time is equal to 2400 s (16$t_e$). The time step is variable with a CFL condition of 0.8 in order to maintain a stable simulation, and this results in average time steps of 0.02 and 0.01 s on meshes I and II respectively. In order to justify the use of the no-slip condition it is verified that the first grid point off solid boundaries is inside the viscous sub-layer. The distribution of the dimensionless wall distance $z^+=u^*\Delta z/\nu$, where $u^*$ is the friction velocity, along the bottom wall of the channel at *x*/*L* = 0.8 through the first and second compartments is presented in Fig. 3. It shows that for meshes I and II the maximum values of $z^+$ are approx. 10 and 7, respectively and hence both simulations are considered wall-resolved LES [15].

## Results and discussion

### Description and validation of the flow hydrodynamics

#### Mean flow hydrodynamics

**Fig. 4:**
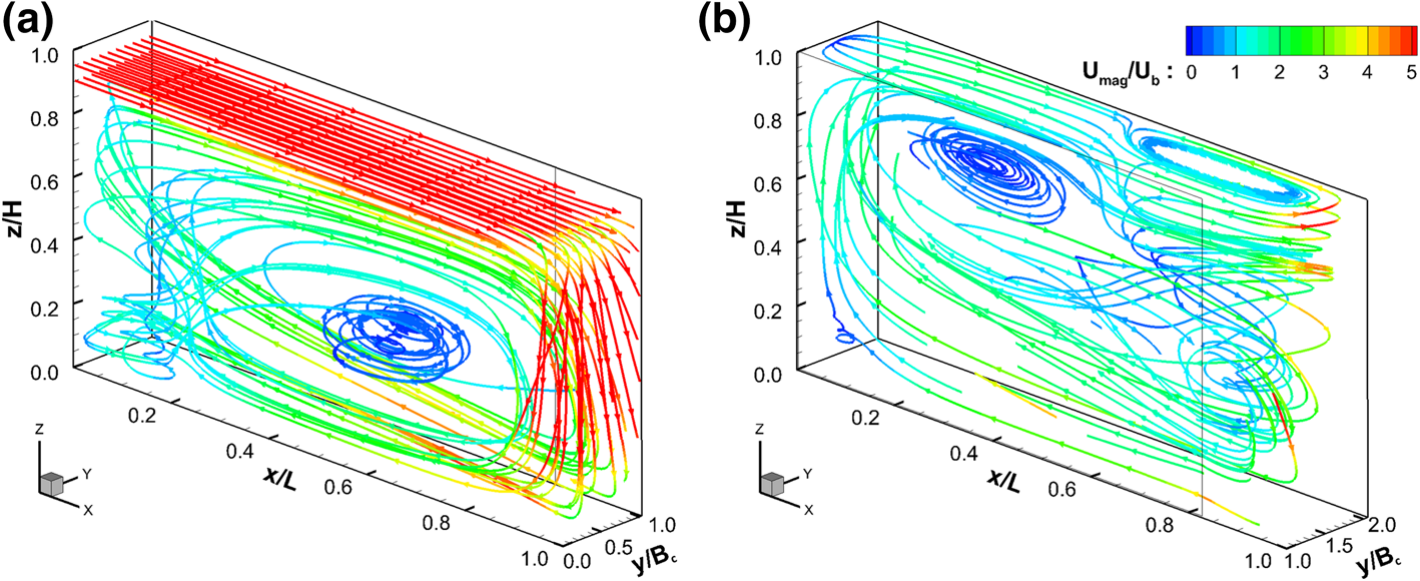
Three-dimensional streamlines coloured with the mean velocity magnitude, $U_{mag}=\sqrt{\overline{U}^2+\overline{V}^2+\overline{W}^2}$, normalised by $U_b$ of compartment 1 (**a**) and 2 (**b**). Note main flow direction is from left to right in (**a**) and from right to left in (**b**)

The flow developed within the two first compartments of the domain is illustrated through 3D streamlines presented in Fig. 4. The inlet velocity is $U_0\approx 10U_b$ and hence a free-surface jet develops and persists until the opposite wall of the compartment, where some fluid is deflected and recirculates thereby creating a large vortex that occupies a significant portion of the first compartment (Fig. 4a). This large-scale flow structure dominates the flow field from $x/D>0.25$ and has a clockwise rotation. As the jet encounters the opposite wall fluid is guided towards the bottom of compartment 2. The time-averaged flow in compartment 2 is visualised in Fig. 4b where two subsequent vortices develop in the horizontal and vertical direction, the first one (horizontal) near the surface and the battle edge and a result of flow separation (at baffle edge) and the second one (vertical) on the upper half and downstream side of the channel developed as a result of the high-velocity near-bed flow as deflected from the end of compartment 1.

**Fig. 5:**
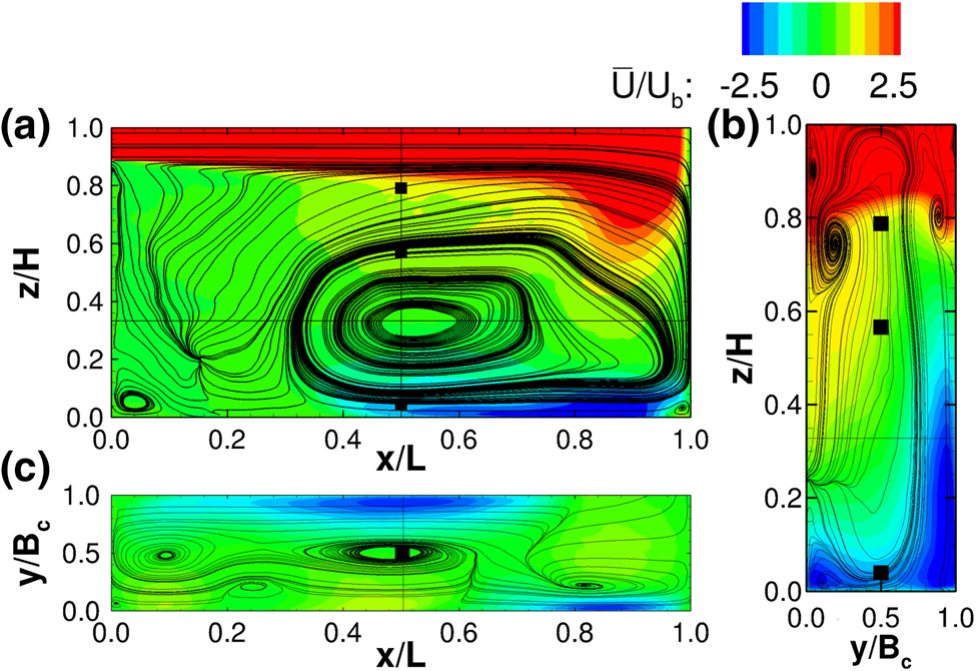
Contours of normalised time-averaged x-velocities $(\overline{U}/U_b)$ with two-dimensional streamlines from the first compartment. **a** y-planes at $y/B_c$ = 0.5, **b** x-plane at *x*/*L* = 0.5 and **c** z-planes at *z*/*H* = 0.35

**Fig. 6:**
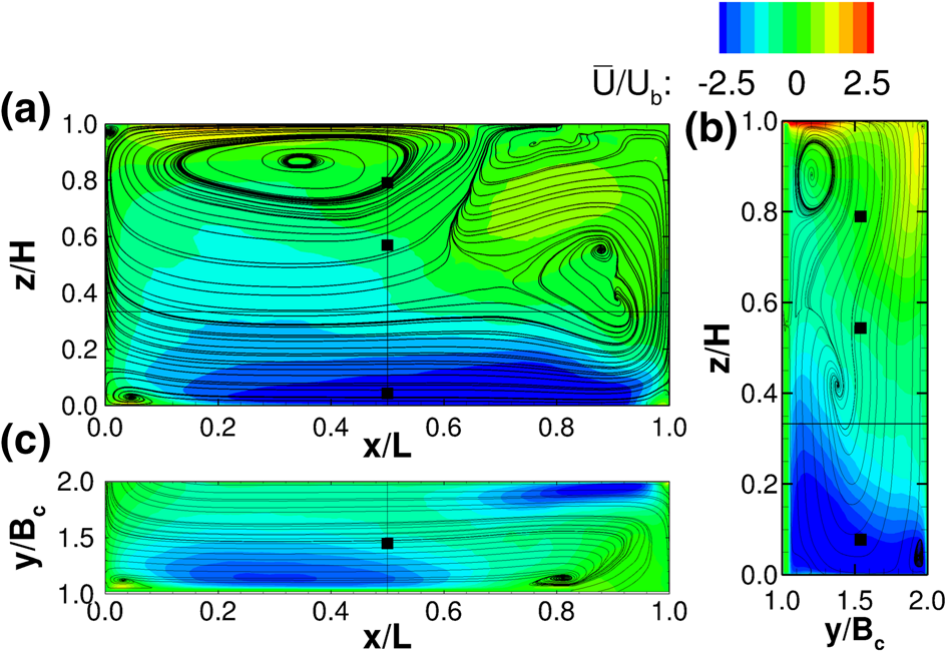
Contours of normalised time-averaged x-velocities $(\overline{U}/U_b)$ with two-dimensional streamlines from the second compartment. **a** y-planes at $y/B_c$ = 0.5, **b** x-plane at *x*/*L* = 0.5 and **c** z-planes at *z*/*H* = 0.35

Flow patterns can also be appreciated from Figs. 5 and 6 which show streamwise velocity contour plots in x-, y- and z-planes. Essentially, due to the impact of the jet upon the compartment wall at *x*/*L* = 1.0 the streamwise flow separates into three distinct directions. The first one remains in compartment 1 and feeds the quasi-vertical recirculation zone seen in Fig. 5a. The second feeds the quasi-horizontal vortex in the first half of compartment 2. The third, drives the flow underneath the horizontal vortex towards the second half of compartment 2 and beyond.

The longitudinal flow pattern in the centreline of compartment 2, i.e. at $y/B_c$ = 1.50, can be described as the inverse of compartment 1 at $y/B_c$ = 0.50 (see Fig. 6). The primary flow path is in this case at the bottom of the channel and induces a subsequent flow separation as it turns towards the downstream compartment. Interestingly, another quasi-horizontal secondary cell develops centred approximately at $x/L=0.35$ and $z/H = 0.85$ as illustrated in Fig. 6a. This vortex occupies the top second half of the compartment and is contained by the prevalent horizontal recirculation zone on the opposite side of the compartment located from approximately *x*/*L* = 0.65 onwards. The velocity magnitude in compartment 2 along the main flow path is markedly reduced, as the momentum of the jet is dissipated by the impact against the side and baffle walls.

#### Turbulent flow hydrodynamics

**Fig. 7:**
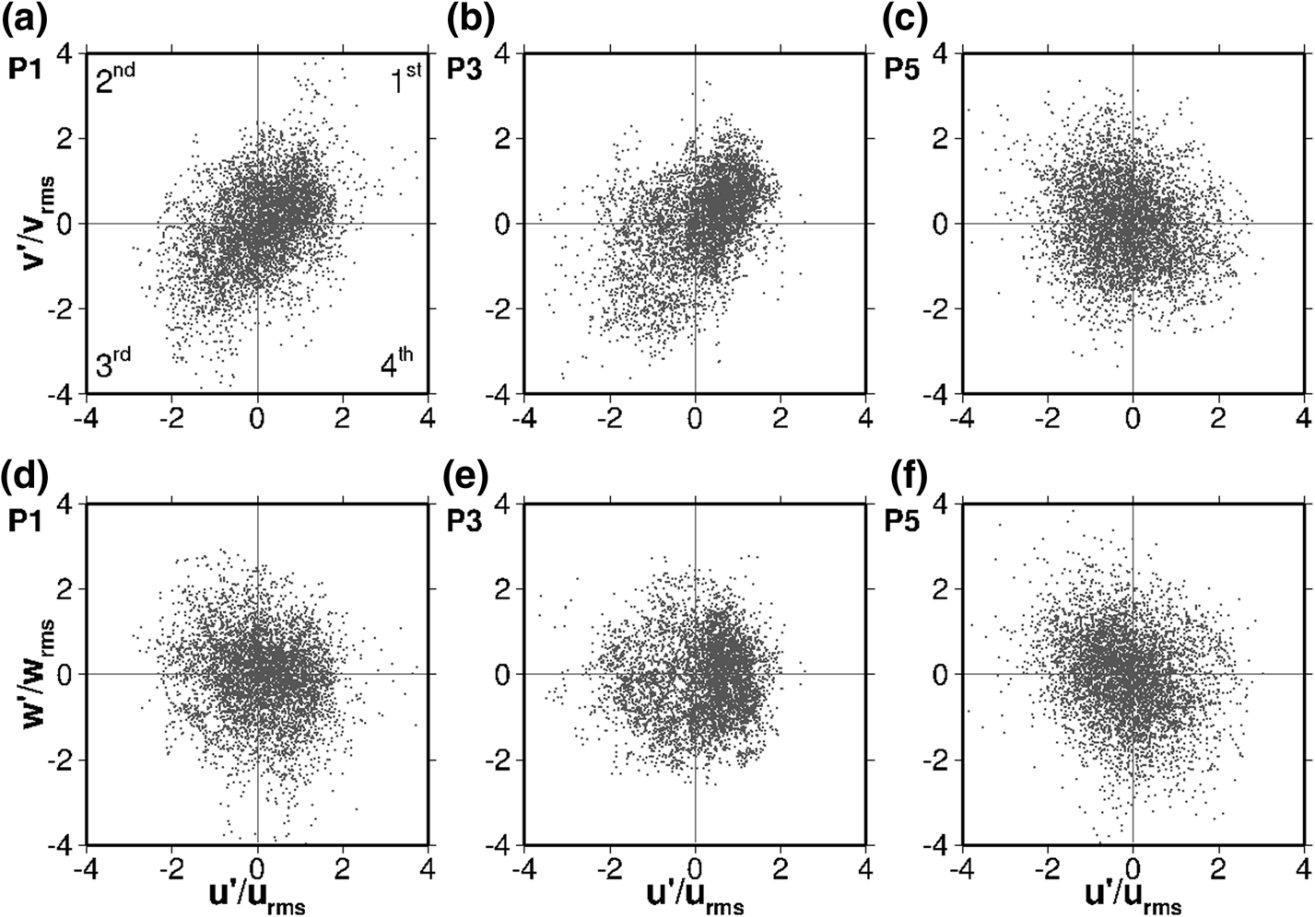
Quadrant plots in the axis $u'/u_{rms}-v'/v_{rms}$ (top) and $u'/u_{rms}-w'/w_{rms}$ (bottom) at three points in the middle of the first compartment: P1 ($z/H=0.772$; left), P3 ($z/H=0.550$; middle) and P5 ($z/H=0.024$; right)

**Fig. 8:**
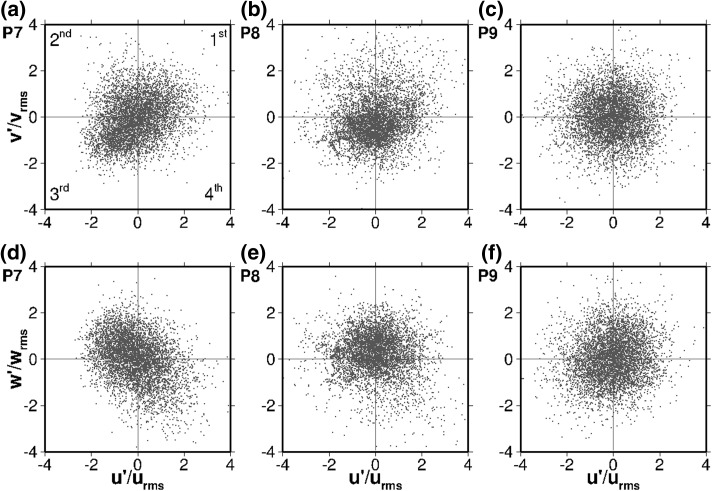
Quadrant plots in the axis $u'/u_{rms}-v'/v_{rms}$ (top) and $u'/u_{rms}-w'/w_{rms}$ (bottom) at three points in the center of the bend between the first and second chambers: P7 ($z/H=0.772$; left), P8 ($z/H=0.550$; middle) and P9 ($z/H=0.024$; right)

**Fig. 9:**
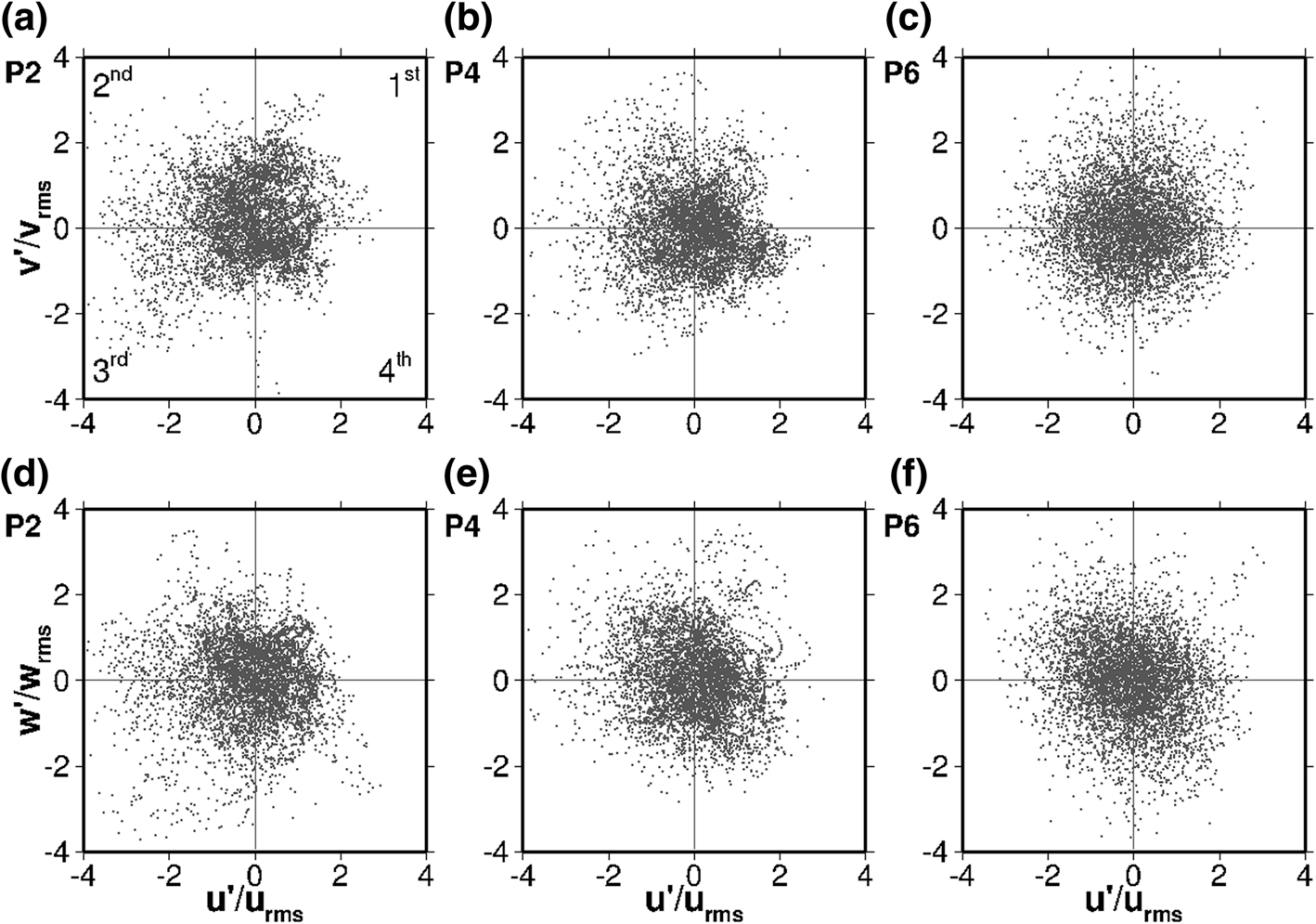
Quadrant plots in the axis $u'/u_{rms}-v'/v_{rms}$ (top) and $u'/u_{rms}-w'/w_{rms}$ (bottom) at three points in the middle of the second chamber: P2 ($z/H=0.772$; left), P4 ($z/H=0.550$; middle) and P6 ($z/H=0.024$; right)

The separated flow creates a high degree of turbulence influencing the hydrodynamics and consequently the passive scalar transport. Quadrant analysis [32] is a technique that helps identifying the presence of coherent structures in the flow and their contribution to the Reynolds stresses. It also illustrates graphically the degree of anisotropy of the turbulent fluctuations and thus is employed to analyse the effect of turbulence on the flow at the nine representative locations depicted in Fig. 2. Figure 7 shows the quadrant analysis plots in the middle of the first compartment at points P1, P3 and P5 as indicated in Fig. 5a and  Fig. 8 presents them in the center of the 180° bend (P7, P8, and P9, Fig. 5b, and finally Fig. 9 focuses on the middle of the second chamber (P2, P4 and P6). In every figure the left column corresponds to the points near the surface (*z*/*H* = 0.772), the sampling locations at half channel depth (*z*/*H* = 0.55) are plotted in the middle column, and the points close to the bottom wall (*z*/*H* = 0.024) are plotted in the right column. For each point the quadrant analysis in both $u'/u_{rms}-v'/v_{rms}$ (top row) and $u'/u_{rms}-w'/w_{rms}$ (bottom row) planes are shown, to account for the significant three-dimensionality of the flow. All the plots are normalised by the root mean squared fluctuations $u_{rms}$, $v_{rms}$, and $w_{rms}$. A Table displaying how many points out of the total fall into each quadrant follows each figure to provide further quantification of the data (Tables 2, 3, 4).

**Table 2:** Relative occurrence of turbulent events in each quadrant for Fig. 7

Plane	Quadrant	P1 (%)	P3 (%)	P5 (%)
$u'-v'$	Q1	36	45	18
	Q2	14	13	31
	Q3	30	23	27
	Q4	20	19	24
$u'-w'$	Q1	26	30	17
	Q2	24	14	32
	Q3	20	22	25
	Q4	30	34	25

Figure 7 shows the normalised velocity fluctuations in the axis $u'/u_{rms}-v'/v_{rms}$ and $u'/u_{rms}-w'/w_{rms}$ at three different depths of the central profile of the first compartment. Each quadrant represents a different turbulent event occurring in the flow. At P1 the great majority of the horizontal velocity fluctuations (Fig. 7a) fall into the first ($u'$ and $v'$ $>0$) and third ($u'$ and $v'$ $<0$) quadrants (36 and 30% respectively, according to Table 2), resulting in an ellipsoidal shape. This suggest a prevalence of the high-momentum motion coming from the inlet. In the vertical $u'/u_{rms}-w'/w_{rms}$ plane (Fig. 7d) the turbulence is more isotropic, as the turbulent events are more evenly distributed among the quadrants. In the lower part of Fig. 7d there is a number of events characterised by rather negative values of $w'/w_{rms}$; these may correspond to the periodic low-frequency oscillations of the upper boundary of the shear layer formed between the jet coming from the inlet and the recirculation zone (see Fig. 12). Those fluctuations at the upper boundary of the main recirculation vortex are key to the entrainment and tracer mixing process, detaching solute from the inflow jet that moves towards the bend and incorporating it into the circulation of the first compartment.

P3 is located at half channel depth and the $u'/u_{rms}-v'/v_{rms}$ plane (Fig. 7b) shows a similar pattern to P1, although the turbulent events are more clustered, especially in the first quadrant (forward-upwards motion is dominant), which comprises 45% of the points. Such difference derives from the fact that P3 is near the core of the large eddy that dominates the first chamber (see Fig. 5a) while P1 is closer to the upper shear layer. As with P1, the fluctuations in the $u'/u_{rms}-w'/w_{rms}$ axis for P3 (Fig. 7e) are much more isotropic than in the horizontal plane, being the most remarkable feature the prevalence of one direction ($u'/u_{rms}>0$, first and fourth quadrants), revealing that P3 is over the core of the recirculation cell most of the time, where the high-momentum current from the inflow is driving the flow.

P5 is in the same horizontal location as P1 and P3, but close to the bottom wall and influenced by its boundary layer. As a result the turbulence is quasi-isotropic, dominated by the small-scale dissipative eddies in spite of the large-scale flow structures. Still, in both planes ($u'/u_{rms}-v'/v_{rms}$ and $u'/u_{rms}-w'/w_{rms}$) the second quadrant exhibits a higher occurrence of turbulent events (31 and 32% respectively), revealing the presence of the recirculation towards the inlet (as illustrated in Fig. 5a, c).

**Table 3:** Relative occurrence of turbulent events in each quadrant for Fig. 8

Plane	Quadrant	P7 (%)	P8 (%)	P9 (%)
$u'-v'$	Q1	26	21	24
	Q2	20	15	26
	Q3	36	35	25
	Q4	18	28	25
$u'-w'$	Q1	17	32	26
	Q2	36	33	23
	Q3	19	17	29
	Q4	27	18	23

Figure 8 presents the normalised velocity fluctuations in the axis $u'/u_{rms}-v'/v_{rms}$ and $u'/u_{rms}-w'/w_{rms}$ for P7, P8, and P9. These sampling locations are in the 180° bend between the first and the second compartments (see Fig. 2). Overall there is a higher scattering of the turbulent events, particularly at half depth (P8), revealing higher multi-directionality as the flow is no longer dominated by such a strong singular large-scale vortical structure as in the first compartment. A high degree of turbulent mixing takes place in the sharp bend. Regarding the horizontal fluctuations at P7 (Fig. 8a), which is close to the surface, there are two interesting features: (1) a higher concentration of points (36%) in the third quadrant, corresponding to flow returning into the first chamber interfering with the wall, and (2) a significant number of events for $v'/v_{rms}>2$ (while very few are below $v'/v_{rms}=-\,2$), representing highly energetic motion towards the second chamber.

Figure 8d clearly shows a higher frequency of events between the second and fourth quadrants (36 and 27% respectively) occurring at P7 in the $u'/u_{rms}-w'/w_{rms}$ plane. These events can be described as downwards flow directed towards the wall (fourth quadrant) and upwards returning flow in the opposite direction (second quadrant). The returning flow is remarkably less turbulent than the incoming one (lower magnitudes of $u'/u_{rms}$ and $w'/w_{rms}$ in the second quadrant), reflecting the energy loss induced by interference with the wall.

P8 exhibits a similar behaviour to P7 in the horizontal $u'/u_{rms}-v'/v_{rms}$ plane (Fig. 8b), although with a higher turbulence intensity and dispersion. There is still a higher concentration of points in the third quadrant (35%), corresponding to flow returning to the first chamber. There are less events in the first and second quadrants, but they exhibit high absolute values of $u'/u_{rms}$ and $w'/w_{rms}$, indicating highly energetic flow motion towards the second compartment occurring at lower frequencies than the returning flow. In the vertical $u'/u_{rms}-w'/w_{rms}$ plane (Fig. 8e), P8 shows high-frequency events in the first and second quadrants (32 and 33% respectively), indicating a prevalence of upwards motion, while there are some very energetic low-frequency events in the third and fourth quadrants, suggesting oscillations of the main vortex core.

The turbulence at P9, which is near the channel bed, again appears more isotropic in nature than on the other two points because of the bottom boundary layer’s influence. In both planes (Fig. 8c, f) there are slightly more turbulent events in the negative side of the $u'/u_{rms}$ axis.

**Table 4:** Relative occurrence of turbulent events in each quadrant for Fig. 9

Plane	Quadrant	P2 (%)	P4 (%)	P6 (%)
$u'-v'$	Q1	26	23	23
	Q2	30	22	26
	Q3	22	24	27
	Q4	22	31	25
$u'-w'$	Q1	24	24	22
	Q2	29	25	30
	Q3	23	21	23
	Q4	24	29	26

Figure 9 presents the normalised velocity fluctuations in the axis $u'/u_{rms}-v'/v_{rms}$ and $u'/u_{rms}-w'/w_{rms}$ in the middle of the second compartment (P2, P4 and P6). At P2 the second and third quadrants show occurrence of highly turbulent events in both the horizontal (Fig. 9a) and vertical (Fig. 9d) planes. These events represent oscillations of the core of the main recirculation eddy within the second compartment, which is very close to the location of P2.

At P4 the high-turbulence events in Q2 and Q3 as observed in P2 are less accentuated as P4 is further away from the core. The vortex on the right generates high frequency motion towards the bend and downwards, represented in the fourth quadrant of both $u'/u_{rms}-v'/v_{rms}$ and $u'/u_{rms}-w'/w_{rms}$ planes (31 and 29% respectively).

Regarding P6, the turbulence is quite isotropic in the horizontal plane (Fig. 9c). The $u'/u_{rms}-w'/w_{rms}$ plot (Fig. 9f) shows a higher density of events in quadrants 2 and 4 (30 and 26% respectively). This reflects oscillations between a downwards inflow coming from the bend (fourth quadrant) and an upwards recirculation (second quadrant). The occurrence of this upwards motion may be related to the fluctuations in the extent of the main recirculation vortex, similarly to the observed behaviour in the first compartment (see Fig. 7). Again the unsteadiness of the boundaries of the large eddies is key to the solute entrainment and mixing process.

**Fig. 10:**
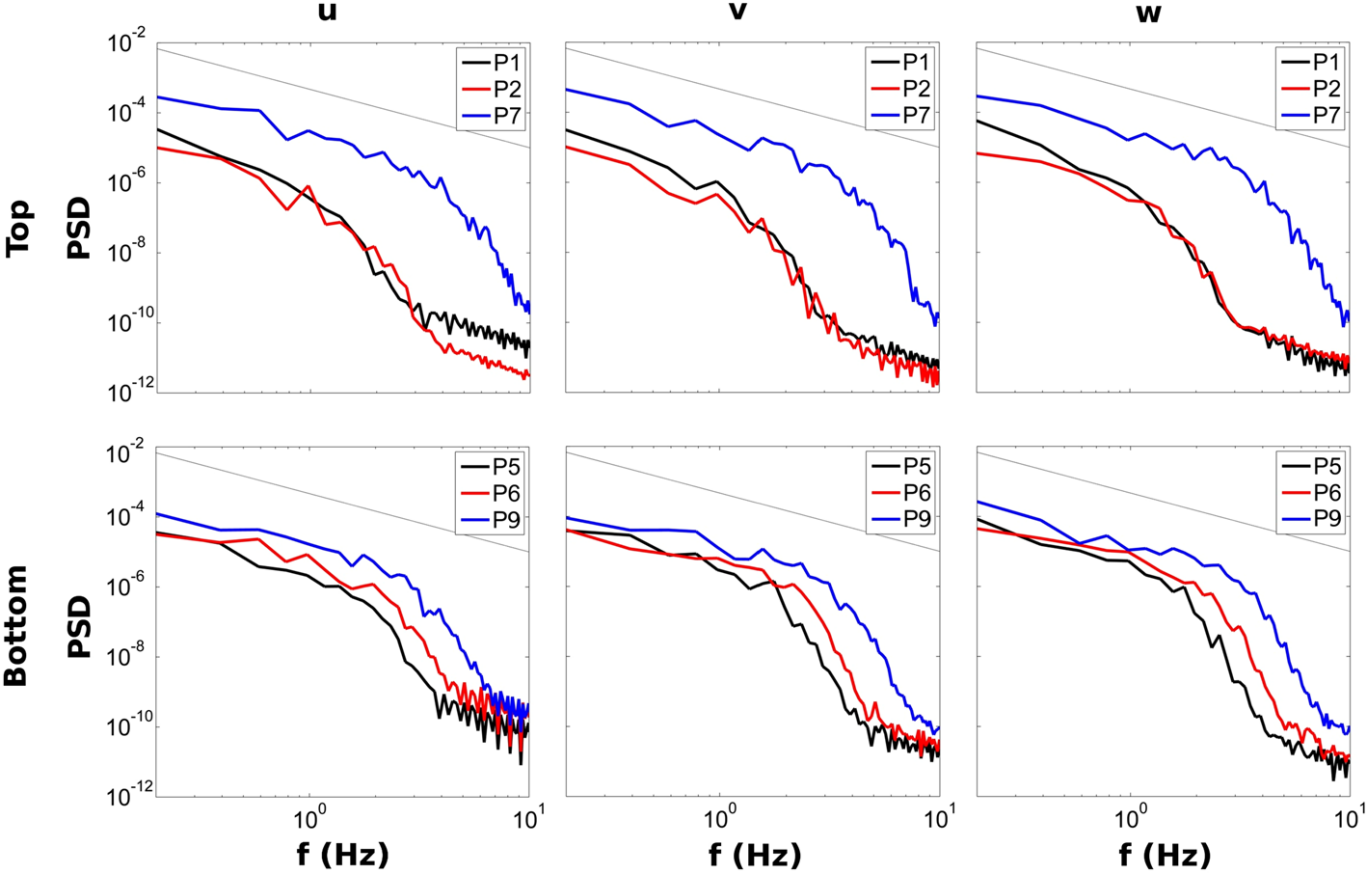
Power Density Spectra of the u (left), v (center) and w (right) velocity components at points 1, 2 and 7 (top) and 5, 6 and 9 (bottom)

Figure 10 presents the velocity spectra of six of the nine points selected for quadrant analysis. The top row corresponds to the points located near the surface (P1, P2 and P7) and the bottom row to the ones near the channel’s bed (P5, P6 and P9), as depicted in Fig. 2. The thin black line represents the − 5/3 Kolmogorov Law, as a reference to homogeneous turbulence decay. The locations at the bend (P7 and P9) exhibit a greater degree of turbulence than the others. However, while the points at the upper half of the domain show a very distinct behaviour in the compartments (red and black lines) than in the bend (blue line), the patterns are more homogeneous near the bottom wall. Noteworthy is the fairly similar behaviour of the points in the centre of both compartments (P1 vs. P2 and P5 vs. P6). P6 seems to carry a bit more energy than P5 and its decay starts at slightly lower frequencies, probably due to the fact that, unlike the first compartment, most of the momentum in the second chamber is advected through the lower half of the compartment. The majority of the spectra in Fig. 10 seem to capture rather well the inertial subrange of the turbulence cascade which follows closely the canonical −5/3 slope. However, the spectra at P1 and P2 (especially the former) exhibit a steeper slope before the rapid decay in the viscous range. It is interesting to relate this anomaly with the fact that the nature of turbulence at these two points is rather anisotropic (see Figs. 7, 9).

#### Validation of the three-dimensional velocity field

**Fig. 11:**
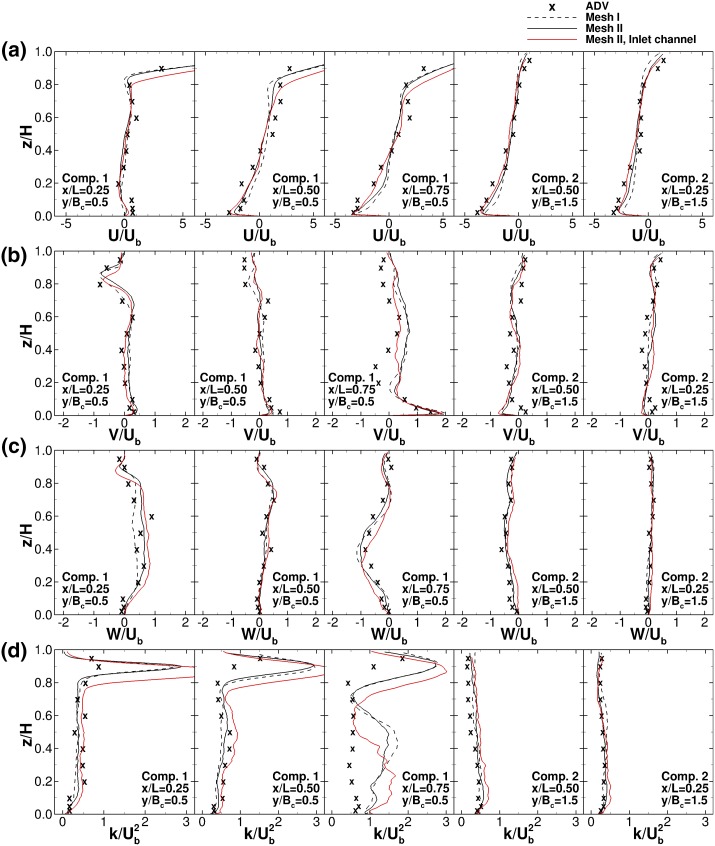
Vertical profiles of normalised **a** horizontal, **b** transverse and **c** vertical velocities and **d** turbulent kinetic energy in compartment 1 at *x*/*L* = 0.25, 0.50 and 0.75, and in compartment 2 at *x*/*L* = 0.50 and 0.25

Validation of the predicted channel hydrodynamics is performed by comparing simulated with measured velocity and turbulent kinetic energy (TKE) profiles which are presented in Fig. 11 at five verticals located along the centreline of compartments 1 and 2, i.e. at $y/B_c$ = 0.50 and 1.50 respectively. The velocities and TKE are normalised with the cross-sectional bulk velocity $U_b$ and $U_b^2$, respectively. Four datasets are compared: ADV measurements from [5], the numerical predictions using the two meshes (using the 1/7th power law inflow condition) and one from the alternative inflow condition performed on mesh II. The mesh sensitivity is only tested in the setup with the prescribed power-law velocity distribution. The LES results on the two grids produce effectively very similar velocity and TKE distributions, suggesting a satisfactory degree of grid convergence of the time-averaged statistics. Both LESs demonstrate a remarkable agreement with measured experimental data by closely matching the mean velocity components in x-, y- and z-directions.

The hydrodynamics close to the solid boundaries are adequately reproduced, however, close to the area of the flow separation ($x/L = 0.75$, $y/B_c = 0.50$), the transverse velocity $V/U_b$ is somewhat underpredicted. This correlates with an overprediction of $k/U_b^2$ for the same profile. These deviations are confined to that particular location and may be expected given the unsteadiness of the hydrodynamics in the transition between compartments 1 and 2 (Fig. 4). There, the flow is characetrised by a shear layer that features high levels of turbulence develop between the primary flow path and the secondary flow structures as a consequence of the flow deflection. TKE levels peak at this separation region, but quickly dissipate halfway through compartment 2 as seen in Fig. 11d where both experimental and LES results show excellent agreement. Another major source of turbulence is due to the shear layer between this high momentum near surface flow and the large-scale vortex below it, previously discussed by [37]. This can be clearly observed in the turbulence profiles of compartment 1 in Fig. 11d.

The choice of inflow boundary condition is not straight-forward due to the lack of flow measurements in the inlet channel of the physical experiment and the presence of the honeycomb flow straightener. As can be seen in Fig. 11 the velocity profiles from the simulations with two different inflow conditions are quite similar in the recirculation area of compartment 1, i.e. $z/H\le 0.8$, and in compartment 2. In the area of the inflow jet, i.e. seen in the first two velocity profiles, there is an apparent downward shift of the peak in the streamwise velocity and TKE profiles of the simulation with precursor channel inflow condition in comparison with the profiles of the simulations which had a power law inflow condition. Also this downshift of high streamwise velocities triggers greater TKE peaks, due to the presence of a stronger shear layer. However, magnitude and location of these peaks do not match the experimental data. Streamwise velocities and TKE profiles in the rest of the domain and all profiles of the spanwise and vertical velocity are not affected by the inflow boundary condition. These results suggest that the use of a prescribed power law velocity distribution at the inlet is most appropriate and hence in the following only results from this simulation on the fine mesh are shown below.

### Passive scalar transport analysis and validation

A particularity of the case under analysis in this paper is the transient nature of the passive scalar dispersion given the small injection time. The unsteadiness of the turbulent flow dominates the scalar transport in the present scenario and thus the instantaneous turbulent fluctuations play a key role in the mixing processes [30, 48]. This is significant for separated flows featuring unsteady vortical structures that last for a certain period of time, trapping and releasing the scalar within recirculation zones. In the simulations performed, a passive scalar is injected at the inlet over a time period of $t_i = 10$ s with an initial concentration of $C_0 = 1$, in analogy to the laboratory experiments. The fate of the scalar along the first and second compartments is firstly described and analysed in relation to the hydrodynamic patterns detailed in previous sections. Secondly, the budgets of the passive scalar fluxes and the relative weigh of the advection and diffusion processes are quantified and discussed. Finally, computed scalar transport in the form of breakthrough curves are compared with the experimental data of [4].

#### Instantaneous tracer transport

**Fig. 12:**
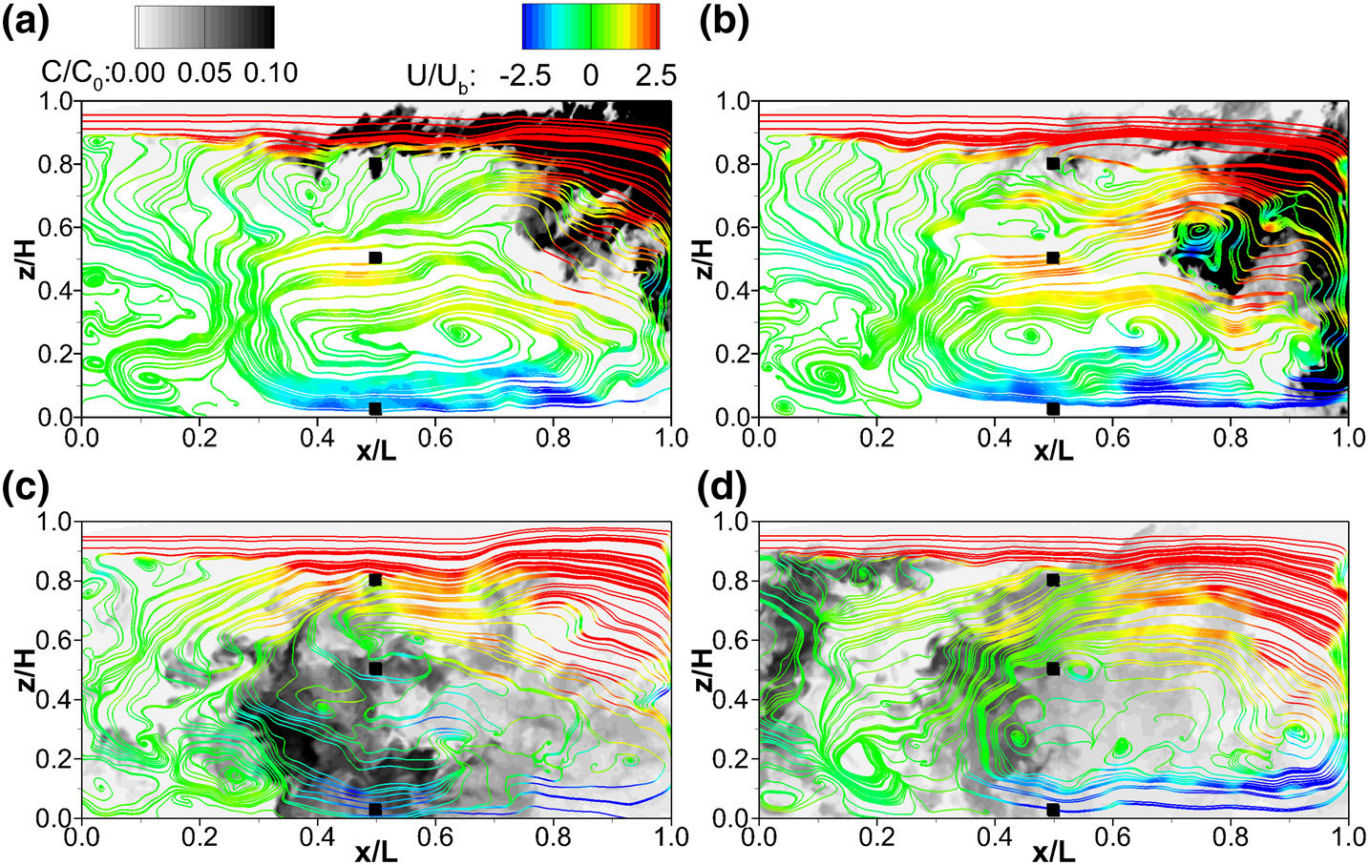
Normalised tracer concentration contours ($C/C_0$) and super-imposed flow streamlines colour-coded by the instantaneous normalised x-velocity ($U/U_b$) in the middle of the first chamber $(y/B_c = 0.5)$ at four different instants: **a** $t = 27$ s $(\theta =0.021)$, **b** $t = 36\,s (\theta =0.028)$, **c** $t = 102\,s (\theta =0.08)$, and **d** $t = 124.5\,s (\theta =0.099)$. Black squares represent the sampling points

The transport process of the scalar over the backwards facing step is analysed based on its instantaneous evolution. The unsteadiness and highly turbulent nature of the flow, previously acknowledged in Sect. 3.1.2, together with the short injection duration of the scalar makes the solute transport dependent on the instantaneous flow field. The scalar transport within the first compartment is visualised in Fig. 12, which presents contours of normalised instantaneous tracer concentration ($C/C_0$) and 2D streamlines coloured by normalised instantaneous x-velocities ($U/U_b$) plotted at the compartment’s midspan plane, i.e. $y/B_c$ = 0.5, at four instants in time, namely $t = 27$, 36, 102 and 124.5 s, for which $t = 0$ s when the passive scalar is injected at the compartment inlet plane.

Figure 12 illustrates the turbulent nature of the flow in the first chamber. As seen in the time-averaged flow field exhibited in Fig. 5a, the flow is dominated by the inflow jet on the upper side of the chamber, a recirculation zone (in the form of a large-scale) eddy underneath it and a small counter-rotating vortex at the bottom left. However, Fig. 12 shows how the boundaries of these two vortices oscillate in time, the formation of several cores at different instants, or the emergence of small rollers at the interface between the inflow jet and the main eddy, which can be related to the low-frequency events reported in Fig. 7d. As an example, the core of the dominating large-scale eddy changes its position in time, as it oscillates between *x*/*L* = 0.4 and 0.7 together with an irregular sudden subdivision into two or more cores, as shown in Fig. 12b, d. Additionally, Fig. 12 also illustrates the transient nature of the tracer injection and its dependence on the instantaneous flow field, as some transient eddies trap and advect high concentrations of tracer. Following the evolution in time, Fig. 12a shows the high momentum of the inflow advecting the tracer towards the exit of the first compartment. Due to the instability of the shear layer between the inflow jet and the large-scale recirculation a small fraction of tracer entrains the dominating flow structure before $x/L \approx 0.7$.

Figure 12b suggests that the clockwise rotation of the dominating large-scale eddy transports the majority of the tracer down the end-wall of the compartment towards the channel bottom. The presence of an instantaneous eddy at $x/L = 0.75$, $z/H = 0.6$ is noteworthy since it traps part of the tracer and directs it towards the centre of the compartment. At $t = 102$ s, Fig. 12c, the majority of the tracer is transported halfway through the compartment and continues to be transported towards the entrance of the chamber. It is observed in Fig. 12d that at $t = 124.5$ s part of the tracer is trapped inside a recirculation area directly downstream of the inlet, below the free-surface jet. At this instant the main recirculation zone occupies the right half of the compartment and tracer is accordingly transported and mixed by the clockwise motion of the recirculation.

**Fig. 13:**
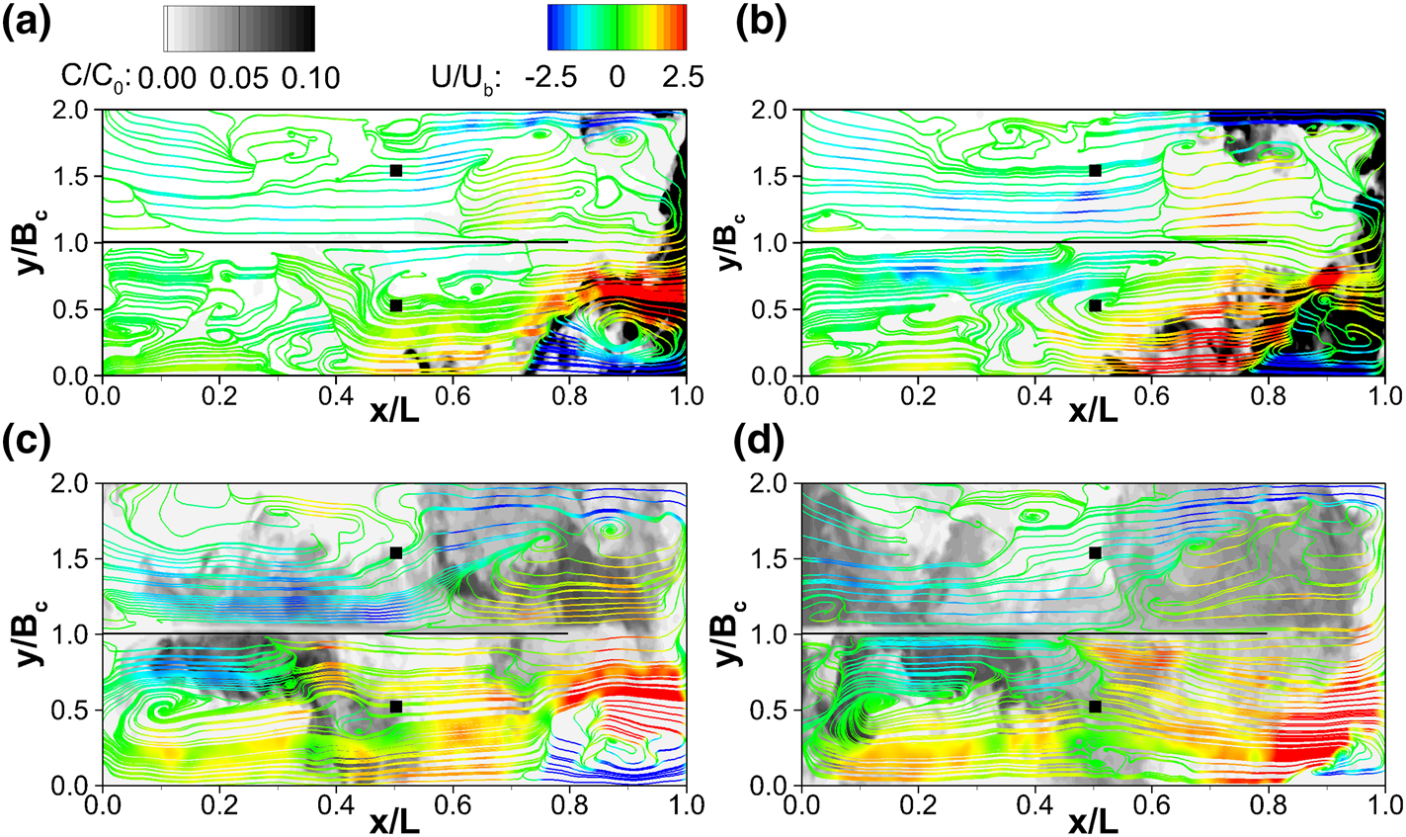
Normalised tracer concentration contours ($C/C_0$) and super-imposed flow streamlines coloured by the non-dimensional streamwise velocity ($U/U_b$) on a horizontal plane ($z/H = 0.65$) extracted at the middle of the first $(y/B_c<1)$ and second $(y/B_c>1)$ chambers. **a** $t = 27\,s (\theta =0.021)$, **b** $t = 36$ s $(\theta =0.028)$, **c** $t = 102$ s $(\theta =0.08)$, and **d** $t = 124.5$ s $(\theta =0.099)$. Black squares represent the sampling points location

Figure 13 shows instantaneous streamlines and tracer concentration contours at the first and second compartments in a horizontal plane at $z/H = 0.65$ at the same instances in time depicted in Fig. 12. As observed in Fig. 5c there is a complex horizontal secondary flow inside the first $(y/B_c<1)$ compartment, dominated by a double-cored large eddy through most of the chamber, excluding the bottom right corner, affected by the downwards flow along the far-end wall ($x/L=1$). The quadrant analysis at P3 (see Fig. 7) revealed strong three-dimensionality and anisotropy subjected to high and low-frequency oscillations of the main recirculation cell. Figure 13 confirms these previous observations by depicting a very transient vortical structure which results in an uneven distribution of the tracer across the compartment’s width. The oscillations at the boundary between the main secondary cell and the counter-rotating downwards jet along the far end-wall (between $x/L= 0.7$ and $x/L=0.9$) have a direct impact on the amount of tracer entrained in the recirculation of compartment 1 versus the fraction that moves into the second chamber. In particular at $t = 36$ s (Fig. 12b) a high tracer concentration appears trapped in the corner recirculation of compartment 1.

Figure 6c shows the second chamber $(1<y/B_c<2)$ is dominated by only one recirculation cell. The transient flow field depicted in Fig. 13 confirms the strong three-dimensionality quantified in Fig. 9. The instantaneous turbulence of the second chamber completely determines the fate of the tracer, driving it first towards the baffle at $y/B_c=2$ and recirculating it within the first half of the compartment, while another portion of the tracer is advected towards the third chamber along the interior wall $(y/B_c=1)$. Hence the tracer fate on this plane of the second compartment provides a clear example of how inefficient would be to derive the scalar transport from the time-averaged flow (Fig. 6c) given a short-timed injection as the one under analysis in this work. Generally, there is a strong interconnection between streamlines and tracer contours; areas of high tracer concentration coincide with regions of fast fluid movement, i.e. streamlines coloured in red or blue, suggesting that the high momentum fluid advects the passive scalar (Fig. 12a, b).

**Fig. 14:**
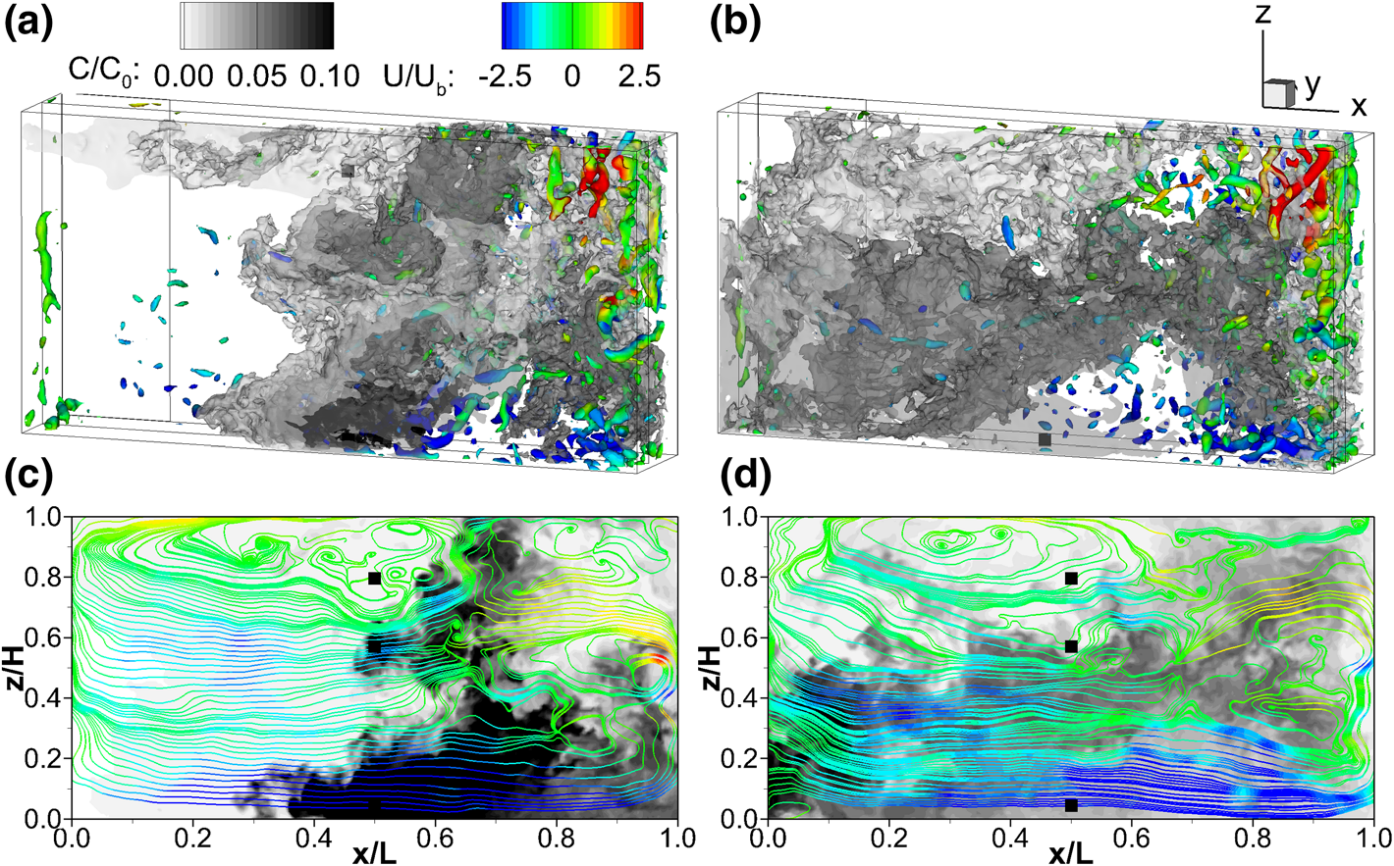
Instantaneous flow field and tracer transport in the second compartment. Top: three-dimensional iso-surfaces of turbulent structures (Q = 1) and tracer concentration ($C/C_0=0.1$); bottom: solute contours and velocity streamlines centreline plane at $y/B_c$ = 1.50; left (**a** and **c**) $t=63$ s $(\theta =0.05)$; right (**b** and **d**) $t=102\,s (\theta =0.08)$

Scalar transport along the second compartment is shown in Fig. 14 with iso-surfaces of scalar concentration (grey-shaded) and the Q-criterion (coloured by the streamwise velocity), a method commonly used to educe turbulent structures [23] (Fig. 14a, b). This is accompanied by two longitudinal planes showing tracer concentration contours and instantaneous streamlines (Fig. 14c, d). At $t=63s$, the highest scalar concentration is advected towards the channel’s bottom (Fig. 14a, c). At $t=102$ s the tracer is more mixed, but is mainly transported along the lower half of compartment 2. The lower half of the compartment, i.e. $z/H \le$ 0.5, is characterised by flow uniformity and low turbulence intensities. The quadrant analysis provided evidences of such isotropy in the turbulence with the analysis at P4 and P6 in Fig. 9. The turbulent structures are mainly located in the vicinity of the 180° bend, i.e. in the transition from compartment 1 to 2, where a counter-clockwise recirculation zone is present (see Fig. 6). In general, the small turbulent structures correspond to rollers generated at the core of the recirculation zones or shear layers, where the tracer concentration is low. The passive scalar is advected by the high-momentum currents and the higher tracer concentrations are located there.

#### Scalar transport fluxes analysis

During visualisation and analysis of the instantaneous scalar concentration it appears that advection processes are dominating the scalar’s transport. The transport of the scalar in the first two compartments is transient and rather chaotic due to the short injection period together with the highly turbulent three-dimensional velocity field and hence very difficult to predict and to analyse. In order to quantify the contributors (advection and turbulent diffusion) to the transport process each term on the RHS of the following scalar transport equation is computed. Note that the short tracer injection time makes impossible to obtain reasonably converged statistics of scalar concentration; therefore $\overline{C}$ and $\overline{u'c'}$ can not be calculated in a reliable manner. As a result, the analysis is based on the instantaneous quantities and given the transient nature of the scalar’s injection, all the transport processes are considered turbulent. The nomenclature used in the following discussion is indicated in Eq. 9 which is based on Eq. 8.

9 $$\begin{aligned} \frac{\partial C}{\partial t} \,\, =\,\, \underbrace{D_t \frac{\partial ^2 C}{\partial x_i^2}}_{Q_{d}}\,\, -\underbrace{ \frac{\partial uC}{\partial x_i}}_{Q_{a,x}}\,\, -\underbrace{ \frac{\partial vC}{\partial x_i}}_{Q_{a,y}}\,\, -\underbrace{ \frac{\partial wC}{\partial x_i}}_{Q_{a,z}} \end{aligned}$$

Here $Q_{a,i}$ denotes advective transport of the scalar with the *i*-velocity component and $Q_{d}$ accounts for the diffusive mass flux (including the SGS contribution). Following [24], the contribution of the sub-grid scales is modelled proportional to the scalar gradient. A reference flux, $Q_0$, is defined in order to normalise the previous mass fluxes and is calculated based on the inflow velocity and a reference concentration such as $Q_0=C_{ref} U_0$. The term $C_{ref}$ is calculated similarly to [22] as,

10 $$\begin{aligned} C_{ref}= \frac{C_0 V}{A U_0} \end{aligned}$$

where $C_0$ is the scalar inlet rate, $V=L H B_c$ is the volume of the first compartment, and $A=d B_c$ is the area of the inflow condition.

**Fig. 15:**
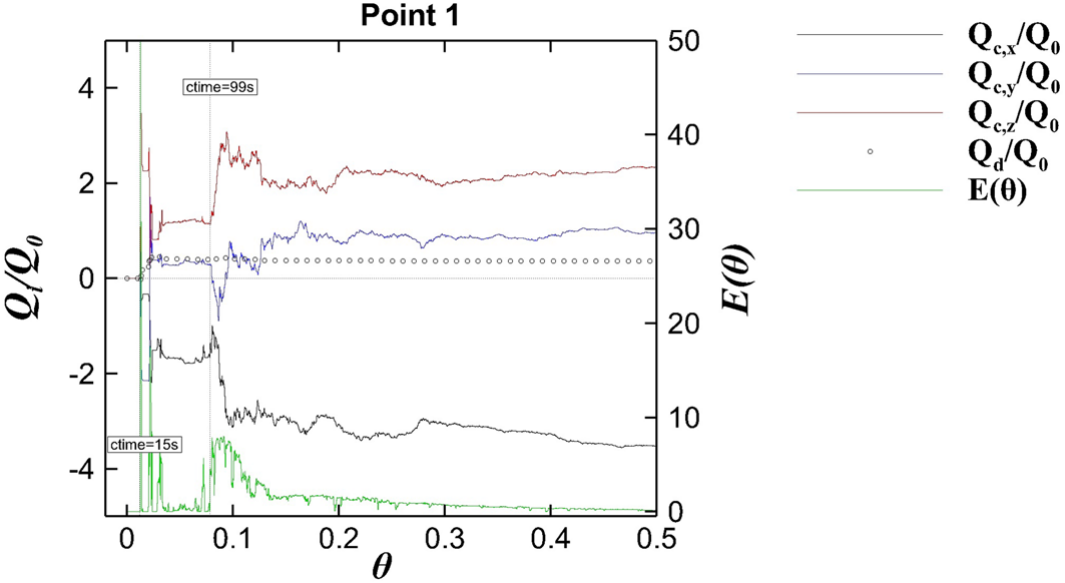
Cumulative curves of convective (black, blue, red) and diffusive (circles) tracer mass fluxes and normalised scalar concentration (green) at P1

Figure 15 shows the normalised cumulative value of mass fluxes defined in Eq. 9 at Point 1, i.e. in the centre of the first compartment, and the normalised instantaneous tracer concentration $E(\theta )$ is superimposed. The normalised time is calculated as $\theta = t/T$, where $T = 1265$ s as the theoretical retention time in the physical model tank for the corresponding experiments [4]. There is a first peak of tracer coming from the inlet at $\theta \approx 0.010$ derived from the initial entrainment of scalar into the recirculating area preciously identified in Fig. 12a, which is later advected towards the end of the first chamber by the streamwise momentum flux $Q_{a,x}$. At $\theta \approx 0.08$ $(t\approx 100\,s)$ a continuous inflow of tracer arrives at P1 mainly through the contribution of the vertical advection $Q_{a,z}$ due to the recirculation process, as depicted in Fig. 12c.

It is obvious that advection via the flow velocity dominates the transport process with the diffusive term an order of magnitude lower, despite the fact that this location is in the middle of the recirculation zone, i.e. where flow velocities are on average very small. However, as identified above the recirculation zone is not steady or stationary and hence bursts of advective transport, here in the form of the peak in the streamwise flux curve, are observed. In contrast, the effect of turbulent diffusion is insignificant and this is also supported by the rather sharp contour boundaries in Fig. 12a, b, i.e. shortly after the injection. In fact, even later, in Fig. 12c, d there are still areas where tracer is absent. In addition, boundaries remain relatively sharp despite the prevalence of steep gradients, i.e. areas where streamlines are bunched together. The very low values of the turbulent diffusion flux is mainly owed to the fact that a very fine grid is used and almost all of the energetic motion of the flow and the scalar is resolved. This is in line with the findings reported in Gousseau et al. [16] who employed LES to study the near field of pollutant dispersion.

**Fig. 16:**
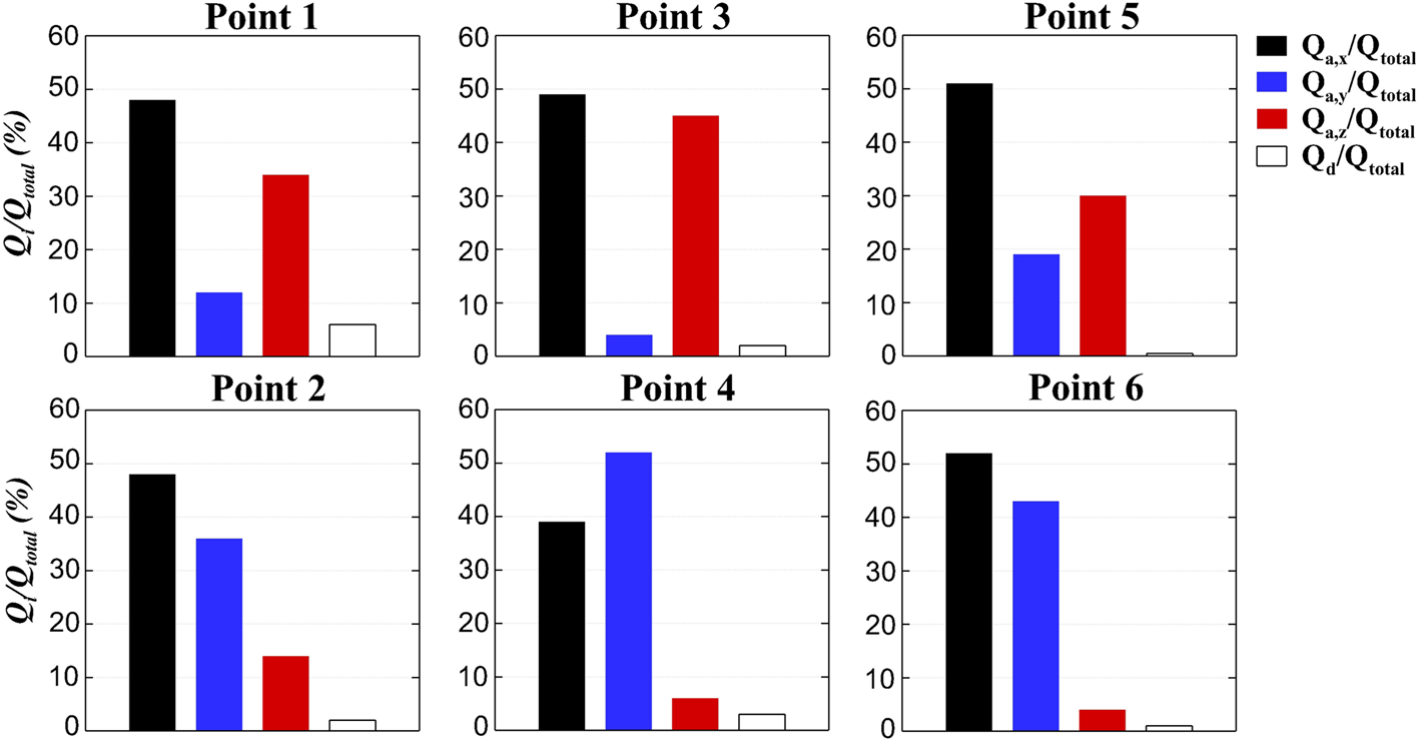
Integral passive scalar advective and diffusive mass fluxes at six reference points as a percentage of the total transport at each location

Depending on the considered location along the backwards facing step, the flow hydrodynamics are different and thus the balance of the mass fluxes driving the scalar transport changes. Figure 16 shows the integral passive scalar mass fluxes as a percentage of the total transport at 3 sampling points in each compartment defined in Fig. 2. The sign of the mass fluxes is eliminated in order to compare their magnitudes, therefore some terms may be net sources or sinks. Results again confirm that the diffusive term is one to two orders of magnitude lower than the advection terms. Another interesting point is that $Q_{d}$ does not respond to the hydrodynamics developed at each point, but merely to the tracer’s concentration magnitude at that location. Hence, the gradient-diffusion hypothesis may not be very reliable to explain the tracers’ fate when concentrations are relatively low or short-term, due to high advection, short injections or a mixture of both, as discussed in [18].

Figure 16 also provides a clear view of the nature of the recirculation in both compartments. The vast majority of the tracer is driven by the recirculation eddies within each chamber. In the first compartment (P1, P3, and P5) the main plane of recirculation is XZ, as demonstrated by the dominance of $Q_{a,x}$ and $Q_{a,z}$. On the other hand, in compartment 2 $Q_{a,x}$ and $Q_{a,y}$ are more relevant, showing a prevalence of the plane XY. This observation is in good agreement with the polar plots of Figs. 7 and 9, which showed an anisotropic distribution of turbulent events in the $u'/u_{rms}-v'/v_{rms}$ (i.e. XY plane) for the first chamber and in $u'/u_{rms}-w'/w_{rms}$ (i.e. XZ plane) in the second, while the dominant planes of recirculation, XZ of compartment 1 and XY of compartment 2, are isotropic.

#### Tracer’s residence time distribution validation

The previous visualisation of tracer contours contributes to an understanding of the scalar’s evolution and the connection with the flow structures and turbulence over the backward facing step. The following residence time distribution (RTD) curves at six different locations in the first and second compartments demonstrate how the normalised instantaneous tracer concentration, $E(\theta )$, varies with the normalised time $\theta$. The significant secondary motion and large turbulent structures combined with the pulse injections of tracer quantities result in transport characteristics that can be strongly dependant on the instantaneous flow field.

**Fig. 17:**
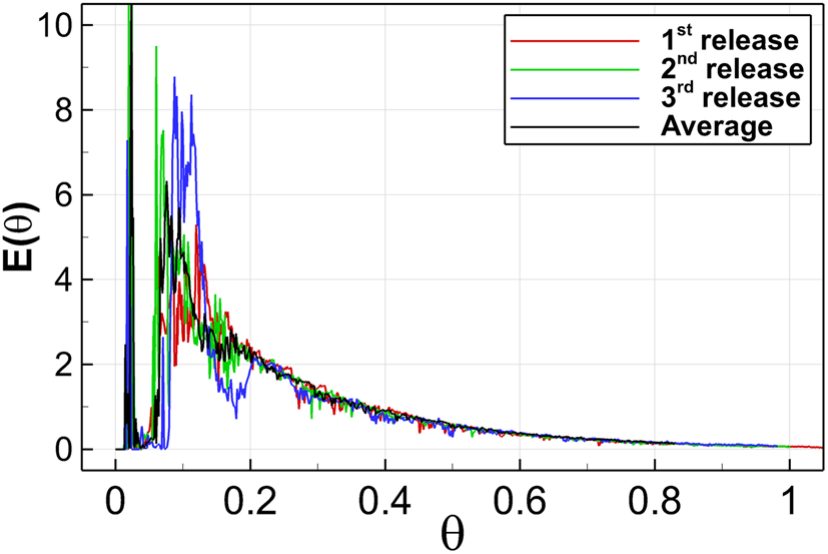
Comparison of the RTD curves obtained at the monitor point P1 between three different releases and the computed average curve

The eddy turn-over time in the first compartment that gives a hint of the periodicity of the flow within the chamber, is significantly higher than the pulse injection time of the tracer ($t_e\approx 150$ s vs. $t_i = 10$ s). Thus, the evolution of the scalar depends strongly on the moment of injection. Figure 17 presents RTD curves obtained at the tracer monitor point P1 from three different injections alongside an average tracer concentration curve of the three releases. Albeit these three curves exhibit some marked differences, they share several features that are related to the dominating flow structure in the first compartment, such as the large concentration peak at $\theta \approx 0.005$, the rapid concentration increase observed from $\theta =0.05$ to $\theta =0.10$, and the exponential concentration decrease beyond $\theta =0.20$. There is a noticeable discrepancy in the instantaneous concentration values between $\theta = 0.10{-}0.20$ amongst the three releases. As Fig. 12d shows, the tracer at $t = 124.5$ s is distributed non-uniformly in the first compartment and its distribution appears to depend strongly on the actual size and location of the instantaneous recirculation areas or the dominating large-scale eddy respectively.

**Fig. 18:**
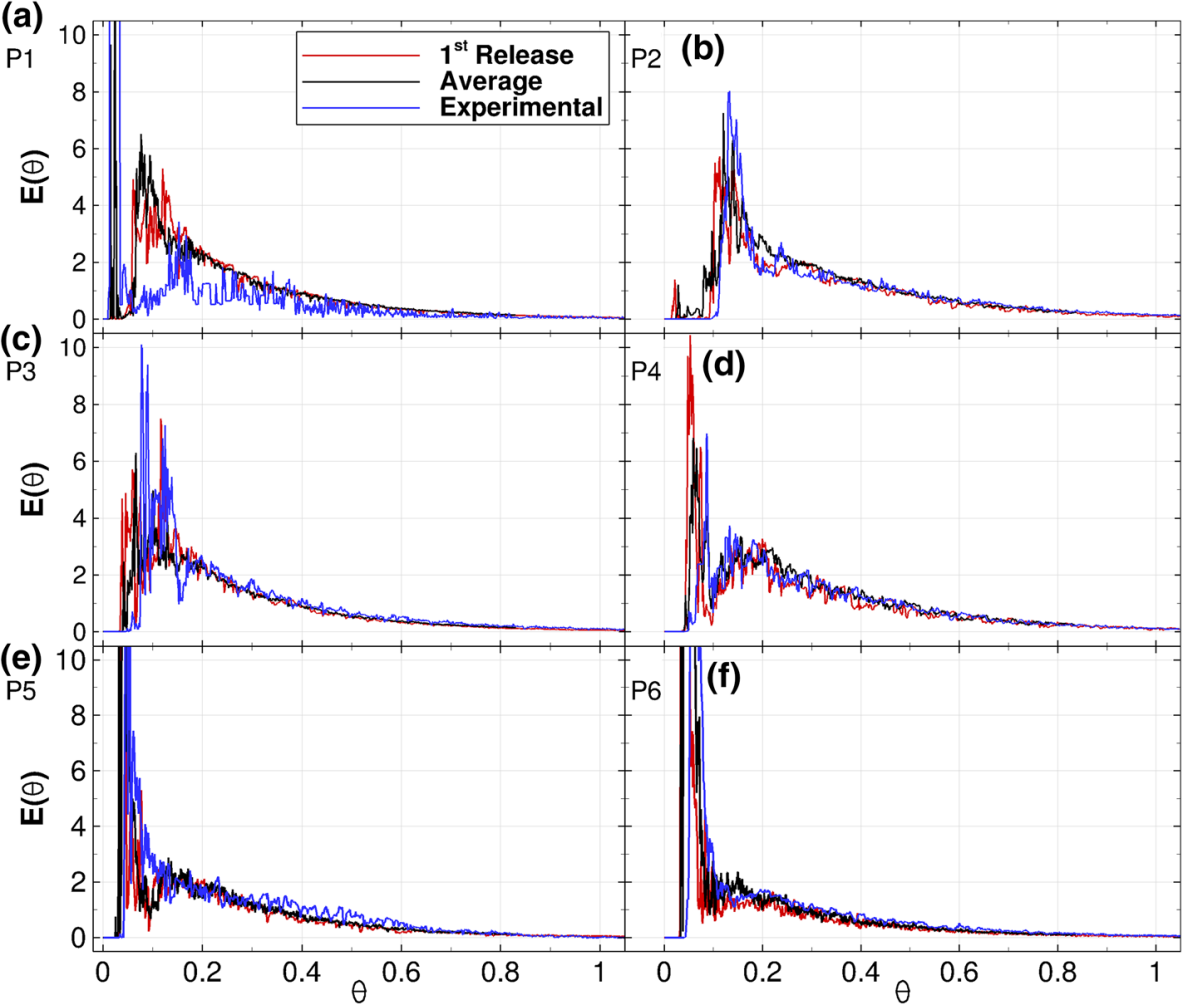
RTD curves obtained from the experiments [4] and the LES of the first injection and the average of three injections at various locations **a** P1, **b** P2, **c** P3, **d** P4, **e** P5 and **f** P6 provided in Fig. 2

Figure 18 presents RTD curves at tracer sampling points P1 to P6 comparing instantaneous tracer data from the first tracer injection simulation, the averaged RTD curve of three separate simulations, and the experimental data. Figure 18a shows computed RTD curves and experimental data at P1. Shortly after the tracer is injected $(\theta \approx 0.01)$ and due to the unstable shear layer between the high momentum inflow water jet and the recirculating flow (see Fig. 12a) some of the tracer is entrained within the flow recirculation area and causes the concentration peak. The LES predicted location of the peak is in good agreement with the experiment. However, for $\theta >0.05$, there is a mismatch between the computed and experimental curves at P1 which can be attributed to the manner tracer is injected in the LES or experiment respectively and the fact that sampling point P1 is close to the tracer injection location. In the LES, the tracer is distributed uniformly over the cross-section and injected for exactly 10s (ideal step distribution), whereas the experimental injection was through a syringe placed perpendicularly to the flow direction. Therefore the data contains a certain degree of uncertainty at the first monitor point which diminishes subsequently in Fig. 18b–f, largely due to turbulent mixing.

The tracer is transported by means of advection to the opposite wall of compartment 1 and is then transported downwards due to the action of the large-scale eddy (see Fig. 12b) only to be then advected backwards along the bottom boundary and finally upwards due to the clockwise rotation of the dominating recirculation zone. This is evidenced by the concentration peak at $\theta \approx 0.05$ at the channel bottom location, P5, Fig. 18e, the peaks around $\theta \approx 0.05$ at mid-channel height, i.e. location P3 (Fig. 18c), and then the secondary peak at P1 around $\theta \>0.1$. After $\theta \approx 0.2{-}0.25$, the RTDs curve exhibit significant tailing, indicating that the scalar is well-mixed within the compartment and is entrapped in recirculation/dead zones from which it is gradually released by virtue of turbulent diffusion.

Figure 18b, d and f show the RTD curves for P2, P4 and P6, located in compartment 2. The short-circuiting at the bottom of the second compartment, outlined previously in Fig. 14c, d, is accountable for the concentration peak at $\theta \approx 0.03$ at P6 and then shortly after a peak occurs at P4, whereas at P2 a concentration peak is not observed until $\theta \approx 0.08$. It can be observed that the LES predicted RTD curves are in good agreement with the experimental data demonstrating that LES is able to reproduce accurately effects of short-circuiting and the role of the large- and small-scale structures onto the tracer’s dispersion.

## Conclusions

The numerical prediction of scalar transport in turbulent separated flows is inherently linked to how turbulence is resolved as flow unsteadiness is a major contributor to scalar dispersion. A flow over a backward facing step (a peculiarity of a chosen contact tank design) has been simulated using the large-eddy simulation method, which is able to resolve the instantaneous flow structures that dominate the flow. The flow patterns in the simulated multi-compartment channel feature pronounced vertical and horizontal flow separation and large-scale recirculating, which have been well captured by the LES. Despite the complexity of the analysed flow, a remarkable agreement between LES and experimental data has been achieved for time-average velocities and turbulent kinetic energy validating the accuracy of the numerical approach.

Quadrant analysis has been employed at nine sampling points located in the domain. This technique has revealed turbulence events and characteristics in agreement with the patterns identified in both the flow and tracer transport analysis. The first chamber is dominated by a strong recirculating eddy that induces anisotropic turbulence with high frequencies events. The bend has exhibited higher multi-directionality and a broader spectrum of frequencies. Quadrant analysis has reflected the energy loss induced in the returning flow after deflection off the opposite wall. The turbulence in the second compartment appears more isotropic in nature than in the first compartment due to the absence of a significant recirculation vortex.

A spectral analysis of the velocity components has also been performed. The points in the middle of both chambers have exhibited a similar spectral energy distribution whereas in the bend a more developed and energetic turbulence cascade has been found especially near the water surface. The two locations that have exhibited stronger anisotropy in the quadrant analysis shared a slope slightly steeper than − 5/3 in the inertial sub-range and entered the viscous sub-range at relatively low frequencies when compared to the other sampling points.

The analysis of the transport of a passive scalar has highlighted the significant role of the instantaneous turbulent flow field on its dispersion, and also that it is extremely sensitive to both large- and small-scale eddies and transient velocity fluctuations of the shear layers or larger vortices. The energetic large-scale structures dominating the compartment’s hydrodynamics govern the advection of the tracer in the entire channel. The presence of intermittent small-scale eddies triggers the tracer’s diffusion transporting it away from the main advective transport routes, however the diffusive flux transport has been quantified as being an order of magnitude smaller than the advective counterpart and this has been quantified via the budget of advective and diffusive terms involved in the passive scalar transport. This is not surprising given the high numerical resolution in both space and time of the LES, hence the turbulent diffusion is only of the order of the sub-grid scale turbulence.

The significant effect of flow unsteadiness on residence time distribution (RTD) curves of a pulse injection has been demonstrated by comparing the RTD curves of three independent scalar injections. The resulting average RTD curve from the different injections predicted by LES has matched remarkably the experimental data owing to the accurate simulation of the relevant instantaneous hydrodynamic characteristics.
